# Species catalogue of *Drymaeus (Mesembrinus)* Alberts, 1850 (Gastropoda: Bulimulidae) from Brazil and new data on morphology and distribution of *Drymaeus (Mesembrinus) interpunctus* (Martens, 1887)

**DOI:** 10.7717/peerj.16037

**Published:** 2023-10-06

**Authors:** Maria Isabel Pinto Ferreira Macedo, Ximena Maria Constanza Ovando, Sthefane D’ávila

**Affiliations:** 1 Instituto de Ciências Biológicas, Museu de Malacologia Professor Maury Pinto de Oliveira, Juiz de Fora, Minas Gerais, Brazil; 2 Programa de Pós-Graduação em Biodiversidade e Conservação da Natureza, Universidade Federal de Juiz de Fora, Juiz de Fora, Brazil; 3 Departamento de Zoologia, Universidade Federal de Juiz de Fora, Juiz de Fora, Brazil

**Keywords:** Land snail, Potential distribution, South America, Peltellinae

## Abstract

**Background:**

Herein, we attempted to obtain detailed data on the distribution of the species of *Drymaeus* (*Mesembrinus*) in Brazil, using biodiversity databases, malacological collections and literature as sources of occurrence records. We provided a catalogue of species, along with distribution maps. We also estimated the suitable distribution of *Drymaeus* (*Mesembrinus*) *interpunctus* using the maximum entropy approach. A detailed description of the anatomy of the soft parts of this species was provided, with new data on the pallial system.

**Materials and Methods:**

For each species we provided information on the compiled data associated with museum collections and the literature. Distribution maps including geographic boundaries, Brazilian biomes and altitude were made with QGIS software 3.16.10 Hannover. For niche modelling, nineteen bioclimatic variables and a topographic variable were used as predictors. The models were performed with MaxEnt version 3.3.3k.

**Results:**

Most of the species are represented by scarce material in malacological collections; for some species, these records correspond to type material, indicating that they have not been recollected. Most of the species were represented by shells making anatomical comparison and DNA analysis difficult, limiting our ability to provide new criteria for species delimitation. Our results allowed us to expand the known distribution area for three species, *Drymaeus dutaillyi*, *D. gereti* and *D. oreades*, with new occurrence records in Brazil. The MaxEnt model showed a thin area of high suitability to *D.* (*M.*) *interpunctus* in the Southeastern Brazil, corresponding to the Atlantic Forest. Minimum temperature of the coldest month and mean temperature of coldest quarter were the variables that most influenced the development of the model.

**Discussion:**

*Drymaeus interpunctus* was described based on specimens collected in Brazil without mention to the exact localities. Herein the new records from databases allowed to expand the known geographic distribution for this species and to infer its potential distribution. Although the type locality of *D. interpunctus* is in Brazil, the anatomy of the soft parts of specimens from this country was not previously described. The anatomy of the reproductive system of the specimens analyzed herein mostly corresponds to a previous description for specimens from Paraguay, except for the absence of penial sheath and the relative length of the bursa copulatrix duct. The results of niche modeling showed a thin area of high suitability for *D. interpunctus* and a vast area of moderate suitability, indicating that this species present a niche breadth that favors its occurrence in a range of different biomes, including less suitable areas.

**Conclusion:**

The small number of records obtained for most of the species and their restricted ranges associated with habitat destruction may indicate that they are of conservation concern.

## Introduction

The family Bulimulidae Tryon, 1867 represents a diverse group of land snails occurring in tropical and subtropical regions of North, Central, and South America (Albers, 1850; Breure, 1979). This family comprises three subfamilies, Bostrycinae *Breure, 2012*, with 15 genera, Bulimulinae Tryon, 1867 (54 genera), and Peltellinae Gray, 1855 (two genera), (MolluscaBase, 2020). Nineteen bulimulid genera are known to occur in Brazil (Salgado & Coelho, 2003; Simone, 2006; Birckolz et al., 2016), including around 120 species. Information on the anatomy of the soft parts is available only for 15 species (Barros Araújo, Rezende & Rodrigues, 1960; Barros Araújo, 1965; Breure & Eskens, 1981; Lanzieri & Almeida, 1964; Lanzieri & Rezende, 1965; Rezende, 1975; Rezende & Lanzieri, 1963). Species numbers are probably underestimated, and new species of bulimulids from different regions in Brazil have been recently described (Salvador, Cavallari & Simone, 2015; Salvador & Simone, 2016; Simone, 2012; Simone & Amaral, 2018).

*Drymaeus (Albers, 1850)* is one of the most species-rich bulimulid genera occurring in Brazil, with approximately 55 species (Simone, 2006; Salvador, Cavallari & Simone, 2015; Simone & Amaral, 2018). This genus comprises two subgenera, *Drymaeus* and *Mesembrinus* (Albers, 1850). The species ascribed to the subgenus *Mesembrinus* are distributed in Central America, South America, and Caribbean. Originally, *Mesembrinus* was proposed as subgenus of *Bulimus* (type species: *Helix (Cochlogena) virgulata* Férussac, 1821). The diagnosis of the subgenus was provided by Albers (1850), and it was originally based on shell characteristics. Pilsbry (1898) considered *Drymaeus* as a genus and *Mesembrinus* as a subgenus, based on shell morphological characteristics. Accordingly, species of the subgenus *Mesembrinus* are characterized by shells with unexpanded, simple outer lips and those from the subgenus *Drymaeus* are characterized by shells with expanded or reflected lips. Breure (1979) added other traits for the differentiation of the two subgenera (*i.e.*: numbers of plates in the jaw and morphology of the radular teeth). The subgenus *Mesembrinus* is characterized by a peristome usually simple, a jaw with more than 20 plates, which are eight times as long as wide, transverse rows of radula V- or W-shaped, with relatively small tri- to multi-cuspid central and latero-marginal teeth. Nonetheless, there is no consensus on the status of the subgenus *Mesembrinus*. Thiele (1929) considered *Drymaeus* and *Mesembrinus* as synonyms, while other authors elevated *Mesembrinus* to genus level (Simone, 2006). Salgado & Coelho (2003) listed all species under *Drymaeus*, without mentioning a subgenus or alternate presentation.

Seven species of *Drymaeus* (*Mesembrinus*) are known to occur in Brazil, *i.e.*: *D.* (*M.*) *dutaillyi* (Pfeiffer, 1857); *D. (M.) gereti* Ancey, 1901; *D.* (*M.*) *interpunctus* (Martens, 1887); *D. (M.) lynchi* Parodiz, 1946a; Parodiz, 1946a; *D.* (*M.*) *oreades* (d’Orbigny, 1835); *D. (M.) puellaris* (Reeve, 1850); and *D.* (*M.) rufolineatus* (Drouët, 1859) (Breure & Eskens, 1981; Salgado & Coelho, 2003; Simone, 2006).

The knowledge on the distribution of *Drymaeus* (*Mesembrinus*) species is fragmentary, the sole contemporary works aimed to list Brazilian species being the checklist provided by Salgado & Coelho, (2003), and the catalog provided by Simone (2006). Among the four species (*i.e.*: *D. (M.) interpuncutus*, *D. (M.) dutaillyi*, *D. (M.) gereti*, *D. (M.) puellaris*) whose type locality is attributed to Brazil, the specific type locality is known only for *D.* (*M.*) *interpunctus* (ie.: Piracicaba, São Paulo state).

Thus, considering that some basic questions, such as where the species occur, remain unanswered, malacological collections may be an important source of information to fill these gaps (Ball-Damerow et al., 2019).

In the present study, we attempted to obtain more detailed data on the distribution of the species of *Drymaeus* (*Mesembrinus*) known to occur in Brazil, using biodiversity databases, malacological collections and scientific literature as sources of occurrence records. From these compiled records, we provided a catalogue of *Drymaeus* (*Mesembrinus*) species from Brazil, along with distribution maps. We also estimate the suitable distribution of *Drymaeus* (*Mesembrinus*) *interpunctus* over the country, using the maximum entropy method. The compiled data associated to the museum deposits allowed to discuss possible constrains to the taxonomic study of these snails. A detailed description of the anatomy of the soft parts of *D. (M.) interpunctus* from Brazil is provided, with new data on the anatomy of the pallial system of this species.

## Material and Methods

### Morphological characterization

The specimens of *D.* (*Mesembrinus*) *interpunctus* were killed and fixed according to Thomé & Lopes (1973), then dissected immersed in ethanol and drawn under a stereo-microscope Olympus^®^, model SZX7 equipped with a drawing tube. All structures of the reproductive and pallial systems were drawn and photographed. Details of the shell were acquired through scanning electron microscopy. The shell was mounted, without any preparation, on a stub covered with adhesive tape of carbon and imaged in a Hitachi low vacuum tabletop scanning electron microscope, model TM3000. The identification of *D.* (*Mesembrinus*) *interpunctus* was based on the species original description (Albers, 1850) as well as on Breure (1979), Breure & Eskens (1981), and Simone (2006).

### Data collection

To provide an annotated checklist of the species of the genus *Drymaeus,* subgenus *Mesembrinus*, an intensive research was performed using data from the literature, malacological collections’ databases and data portals as Global Biodiversity Information Facility, Sys Tax–Zoological Collections and SpeciesLink (GBIF, 2020; SpeciesLink, 2020; SysTax, 2020). We also included records from the malacological collection of Museu de Zoologia da Universidade de São Paulo kindly provided by the curator. All museum deposition numbers are provided in the checklist, including information on type material. The material analyzed in person by the authors are labelled as “material examined”, additionally a list of museum deposits ascribed to each species is provided in the topic “museum deposits”, with the objective of facilitate future studies on taxonomy and morphology of *Drymaeus* (*Mesembrinus*) species. To provide a provisional characterization of the geographic distribution of these poorly known species, we compile and critically analyze all the occurrence records obtained through an intensive search in the literature, biodiversity databases, and malacological collections. We considered species identification in literature records as valid when it felt in the known distribution area of the species. Concerning the occurrence records obtained *via* malacological collections, in some cases we were able to validate species identification using shell morphological criteria currently accepted, because the images of these specimens were available in the databases, they were ceded by the curators of the collections, or we had the chance to analyze the specimens personally. Nonetheless, for some of the records herein compiled we could not validate species labelling. Thus, we discriminated occurrence records for which species identity was confirmed and occurrence records without confirmation of species identity (Table S1). Accordingly, the generated maps allow to critically consider the known distribution of the species. Discrepant occurrence records falling outside the known range of a species were not included in the maps.

For distribution maps, localities of occurrence of the species were georeferenced with Google Earth Pro (https://www.google.com.br/earth/) and GeoLocate application (https://www.geo-locate.org/). The duplicated points and occurrence records without specific localities were excluded from the analysis. The maps were made with QGIS software 3.16.10 Hannover (QGIS Development Team, 2021), and included geographic boundaries, ecoregions and altitude information, obtained from the Brazilian Institute of Geography and Statistics (IBGE: https://www.ibge.gov.br/geociencias/downloads-geociencias.html).

We provided information on the compiled data associated to the museum deposits (*i.e.*: mode of preservation, number of specimens, collection date, and locality), and from the literature (illustrations and occurrence records). The type locality for each species is given in quotation marks as it is stated in the original publication and in the same language used by the author. The type material of each species have been searched consulting the material housed in different collections (photos, original labels) as well as by consulting of the relevant bibliography (Breure, 1979; Breure & Ablett, 2014). We compiled a total of 95 geographic records of *Drymaeus* (*Mesembrinus*) *interpunctus* from museum collections, and scientific articles used in the Ecological Niche Analysis (Table S1). Taxonomic bibliography were obtained mostly from the Biodiversity Heritage Library website (BHL, 2019).

### Ecological niche models

Nineteen bioclimatic variables and a topographic variable (altitude), at a spatial resolution of 30 arc seconds (∼1 km^2^), were used as predictors. These data were obtained from WorldClim (http://www.worldclim.org). We clipped the environmental data layers to the calibration area defined as South America. The models were performed with the software package MaxEnt version 3.3.3k (Phillips, Anderson & Schapire, 2006). The default parameters of MaxEnt algorithm were used, including a maximum of 500 iterations with a convergence threshold of 0.00001 and 10,000 randomly chosen background localities. 100 replicates were run, and a random test percentage option was used with 75% of presence records randomly selected to generate the models, while the remaining 25% was used to test them. The model was computed as “logistic”. This output returns a continuous map with an estimated probability of presence between 0 (no probability of the species presence) and 1 (high probability of presence), which permits fine distinctions between the suitability of different areas modeled (Giovanelli et al., 2008). For each model, the performance was assessed using the method of the area under the curve (AUC) of the receiver operating characteristic (ROC). The AUC represents the probability for the model to score a presence site (test locality) higher than a random background site (Phillips, Anderson & Schapire, 2006; Elith et al., 2006).

## Results

### Annotated checklist of species of *Drymaeus*, subgenus *Mesembrinus* from Brazil

**Figure 1:**
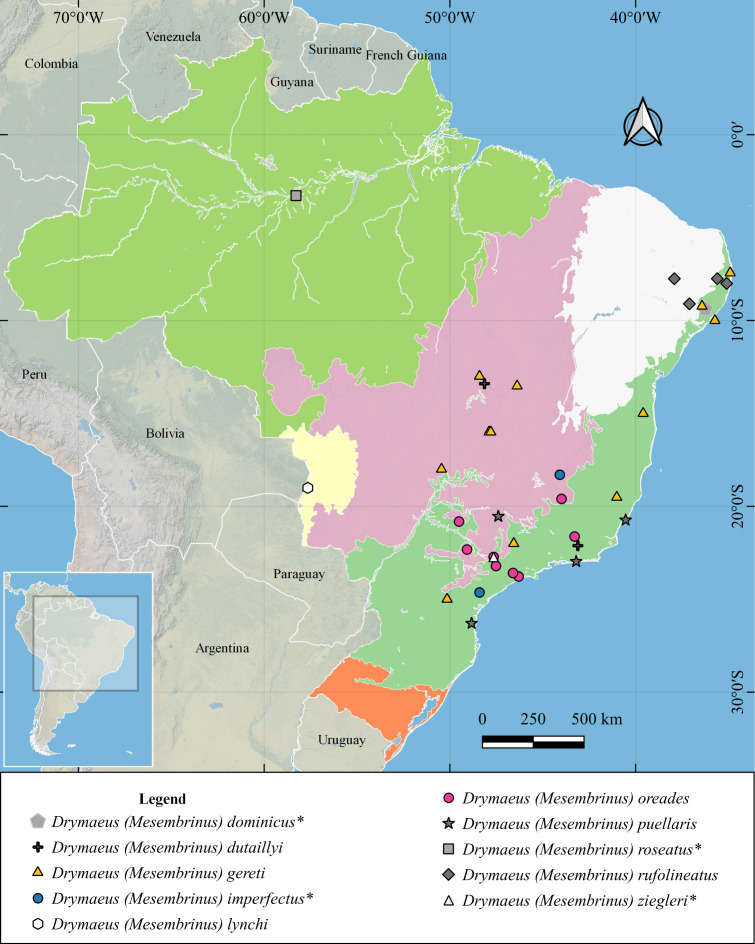
Distribution map showing the biomes occupied by species of *Drymaeus*, subgenus *Mesembrinus,* in Brazil. The species marked with an asterisk (*) correspond to species whose occurrence in Brazil is considered as misidentification cases and doubtful presence.

**Figure 2:**
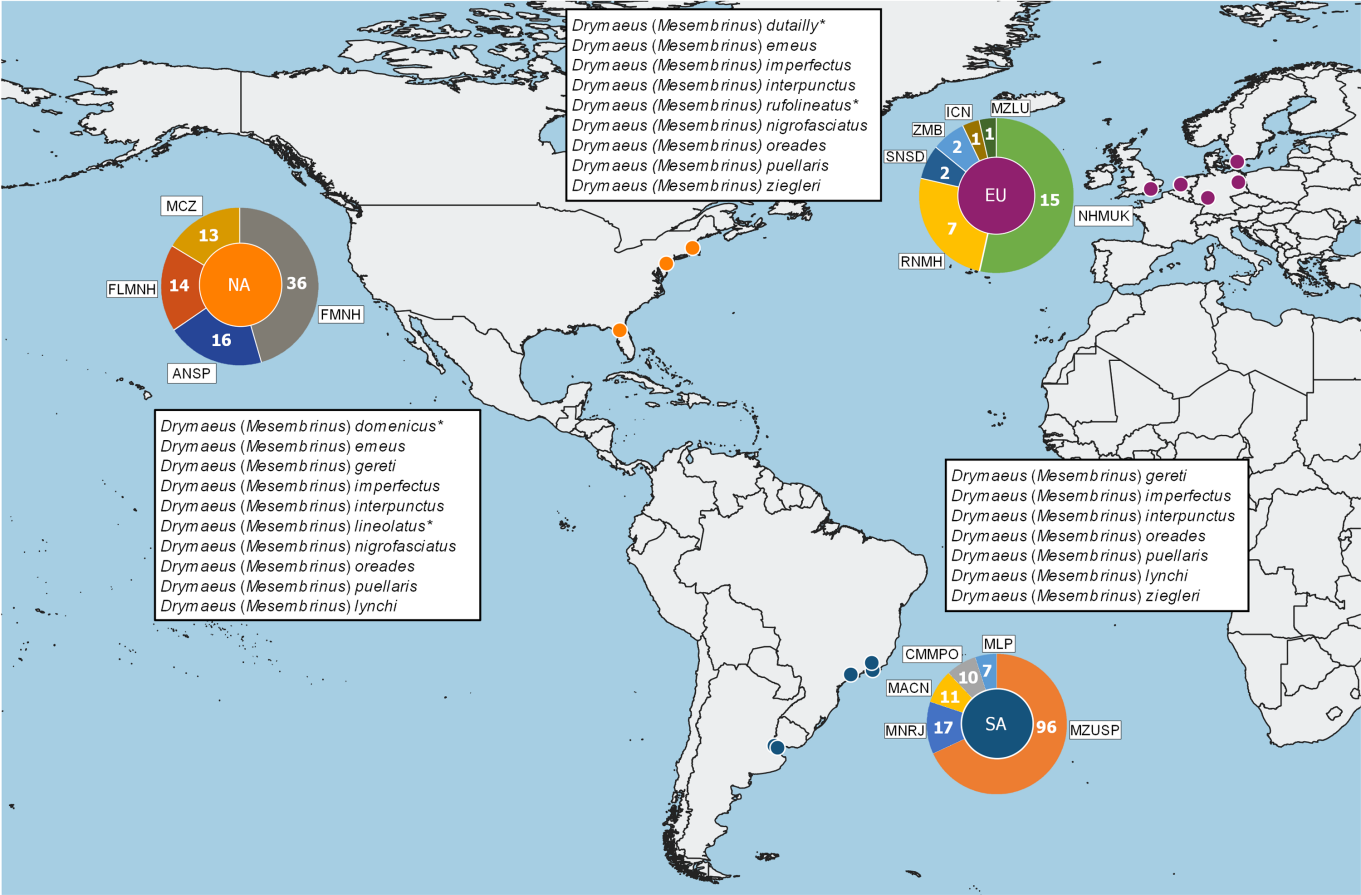
Representativity of *Drymaeus* (*Mesembrinus*) spp. in different malacological collections. Number of lots housed in each malacological collection from Europe (EU), North America (NA) and South America (SA), per species of *Drymaeus*, subgenus *Mesembrinus*. The species marked with an asterisk (*) correspond to species that are present in one collection. Acronyms: ANSP Academy of Natural Sciences of Philadelphia; BMSM Bailey-Matthews National Shell Museum; CMMPO Coleção Malacológica Maury Pinto de Oliveira; FMNH Field Museum of Natural History; ICN Instituto Nacional de Ciencias, Universidad Nacional de Colombia; LMD Löbbecke Museum of Dusseldorf; MACN Museo Argentino de Ciencias Naturales Bernardino Rivadavia; MCZ Museum of Comparative Zoology; MHNG Muséum d’Histoire Naturelle, Geneva; MNRJ Museu Nacional do Rio de Janeiro; MZB Museu de Ciències Naturals de Barcelona; MZLU Lund Museum of Zoology MZSP Museu de Zoologia da Universidade do Estado de São Paulo; RBINS Royal Belgian Institute of Natural Sciences RMNH; Rijksmuseum voor Natuurlijke Historie; SNSD Collection Malakologie –Senckenberg Society for Nature Research; UF Florida Museum of Natural History; MLP Museo de La Plata; ZMB Museum für Naturkunde, Berlin.

A total of 196 occurrence records of seven species of *Drymaeus*, subgenus *Mesembrinus* were compiled for Brazil (Table S1, Fig. 1). Most of the species are represented in malacological collections from North America and Europe (Fig. 2). In some cases, these records correspond only to the type material, ie.: *Drymaeus* (*Mesembrinus*) *dutaillyi* with two records including the lectotype and *Drymaeus* (*Mesembrinus*) *lynchi* with four records, including the holotype and paratype, both in North and South American collections (Fig. 2). Some species whose records in Brazil are uncertain, i.e.: *Drymaeus* (*Mesembrinus*) *dominicus*, *D*. (*M*.) *imperfectus*, *D*. (*M*.) *membranaceus, D*. (*M*.) *nigrofasciatus*, *D* (*M.*) *roseatus* and *D*. (*M*.) *ziegleri*, were not included in the checklist, after verification of data from malacological collections and personal communication of A.S.H. Breure. The records of most of these species in Brazil probably correspond to misidentifications. Thirteen lots housed in different collections lacked information about country and collection site and therefore they have not been included in our analysis. Our results allowed the expansion of the known distribution area for three species, with new occurrence records in Brazil. Herein, we present the first record of *Drymaeus* (*Mesembrinus*) *dutaillyi* for Rio Grande do Sul state, *Drymaeus* (*Mesembrinus*) *gereti* for Alagoas, Bahia, Paraíba, Rio de Janeiro, Minas Gerais, Paraná and Rio Grande do Sul states, and *Drymaeus* (*Mesembrinus*) *oreades* for Minas Gerais state. The distribution maps showing the biomes occupied by the species indicated that all the Brazilian biomes included at least three species. *Drymaeus* (*Mesembrinus*) *interpunctus* is the only species present in all the six Brazilian biomes, mainly in the Atlantic Forest and Pampa. *Drymaeus* (*M.*) *gereti* was recorded in the biomes Atlantic Forest and Cerrado whereas. *D.* (*M.*) *puellaris*, *D.* (*M.*) *oreades*, *D.* (*M.*) *rufolineatus* and *D.* (*M.*) *dutaillyi* were recorded in two biomes.

### Systematic part

**Table:** 

Class Gastropoda Cuvier, 1795
Eupulmonata Haszprunar & Huber, 1990
Stylommatophora A. Schmidt, 1855
Helicina Rafinesque, 1815
Orthalicoidea Martens, 1860
Bulimulidae Tryon, 1867
Peltellinae Gray, 1855
*Drymaeus* Albers, 1850

Type species: *Drymaeus hygrohylaeus* (d’Orbigny, 1835)

Subgenus *Mesembrinus* (Albers, 1850)

*Drymaeus* (*Mesembrinus*) *interpunctus* (Martens, 1887)

*Bulimulus interpunctus* Martens, 1887: 161 (pl.40 fig.6–7)

*Drymaeus interpunctus* —Pilsbry, 1898 [1897–1898]: 287, pl.5, figs. 4, 5. —Ihering, 1923: 191. —Bertoni, 1926: 73; —Haas, 1936: 150. —Haas, 1955: 374. —Parodiz, 1957: 25. —Parodiz, 1962: 43; —Schade, 1965: 217. —Fernández, 1973: 114. —Fernández & Castellanos, 1973: 280. —Quintana, 1982: 95;—Salgado & Coelho, 2003: 162.—Cuezzo, Miranda & Ovando, 2013: 155.

*Drymaeus* (*Mormus*) *interpunctus*—Morretes, 1949: 151.

*Drymaeus* (*Mesembrinus*) *interpunctus*—Miquel, 1989: 81, fig. 13, 16.—Breure & Eskens, 1981: 74, figs. 235–244.—Miquel, 1991: 74.—Köhler, 2007: 150.

*Mesembrinus interpunctus*—Simone, 2006: 145.

*Original description*: Martens, 1887. SitzBer. Ges. naturf. Frde Berlin, p. 161.

*Illustration*: Martens (1881: pl. 40. fig. 6-7, shell), Miquel (1989: figs. 12, 13, 16, shell), Breure & Eskens (1981: figs 235–244, reproductive system), Simone (2006: fig. 486, shell).

*Type material: Lectotype.* ZMB MOL 69126. *Paralectotype*. ZMB MOL 69127.

*Type locality*: “Piracicaba, Prov. S. Paul, Brasilien” (Martens, 1887).

*Material examined:* **Brazil** **•** 1 specimen preserved in ethanol; Barra Mansa, Rio de Janeiro; D’ávila, S. leg.; CMMPO 11327 **•** 4 specimens preserved in ethanol; Juiz de Fora, Minas Gerais; 2017; Santos, E.O. leg.; CMMPO 11328 **•** Pedro Leopoldo, Minas Gerais; 21 Jan 1963; Leme, J. L.M. leg.; Leme, J.L.M. & N. Papavero cols.; MZSP 015580 **•** 1 specimen preserved in ethanol; Itapetininga, São Paulo; MZSP 49313.

*Museum deposits.* **Brazil** •1 shell and 1 soft part preserved in ethanol; São Paulo state; von Ihering, H. leg.; ANSP 73433 •1 shell and 1 soft part preserved in ethanol; Piquette, São Paulo state; von Ihering, H. leg.; ANSP 73438 •1 shell and 6 soft parts preserved in ethanol; São Paulo, São Paulo state; ANSP 73451 •2 shells; Além Paraiba, Minas Gerais; 1967; CMMPO 3341 •1 shell; Chácara, Minas Gerais; 5 Nov 1967; Paula, M.M.M. leg.; CMMPO 3342 •6 shells; Juiz de Fora, Minas Gerais; Jul 1969; Delage, O. leg.; CMMPO 3346 •3 shells; same locality; Jul 1969; Delage, O. leg.; CMMPO 3347 •5 shells; Fazenda São Fidelis, Juiz de Fora, Minas Gerais; 20 Aug 1967; CMMPO 3348 •3 shells; Vila São Vicente, Juiz de Fora, Minas Gerais; 11 Nov 1967; CMMPO 3349 •1 shell; same collection data as for preceeding; 26 Nov 1968; Delage, O. leg.; CMMPO 3350 •4 shells; Caruarú, Pernambuco; 1988; Lima, M.A. leg.; CMMPO 7310 •1 specimen preserved in ethanol; Barra Mansa, Rio de Janeiro; CMMPO 11327 •4 specimens preserved in ethanol; Juiz de Fora, Minas Gerais; 2017; CMMPO 11328 •1 specimen; FMNH 95394 •2 specimens; FMNH 119245•2 specimens; FMNH 206176 •2 specimens; FMNH 31274 •2 specimens; FMNH 31399 •shell; FLMNH 206176 •specimen preserved in ethanol; São Paulo, Avanhadava; MACN 11804 •1 shell; São Paulo, Perus; 1897; Leme, J.L.M. ident.; MNRJ 30445 •1 shell; São Paulo; Tostes, L.R. leg. MNRJ 41129 •3 shells; Curitiba, Paraná; Lange, R.; MZSP 016774 •6 shells; Itu, São Paulo; Morretes, L. leg.; MZSP 016775 •2 shells; Lussanvira, São Paulo; 01 Dec 1935; MZSP 016778 •2 shells; Valinhos, São Paulo; Morretes, L. leg.; MZSP 016781 •5 shells; Fábrica, Cubatão, São Paulo; 01 Sep 1950; Morretes, L. leg.; MZSP 016783 •1 shell; Piracicaba, São Paulo; 01 Nov 1952; MZSP 016784 •1 shell; Fazenda Pilar, Cornélio Procópio, Paraná; Sep 1953; Osório, C.A.M. leg.; MZSP 016785 •3 shells; Chácara Macedo, Curitiba, Paraná; 25 Mar 1932; MZSP 016786 •2 shells; Porto Feliz, São Paulo; 03 Jan 1936; Morretes, L. leg.; MZSP 016789 •1 shell; Estrada de Ferro Sorocabana, Jandira, São Paulo; 20 Sep 1949; MZSP 016811 •1 shell; Rondonópolis, 20km from the city, Mato Grosso; Silva, L.C. leg.; MZSP 026434 •8 shells; Cantareira, São Paulo, São Paulo; 1945; MZSP 027655 •4 shells; caverna, São Mateus, Goiás; 01 Jan 1979; Lino, C. leg.; MZSP 026562 •3 shells; Curitiba, Paraná; 09 Feb 1932; Morretes, L. leg., ex. col. Lange de Morretes, no 03; MZSP 027654 •8 shells; Cantareira, São Paulo, São Paulo; 1945; MZSP 027655 •6 shells; Piracicaba, São Paulo; 1945; MZSP 027656 •10 shells; Alto da Serra, São Paulo, São Paulo; 27 Oct 2016; MZSP 027657 •3 specimens preserved in ethanol; Santa Maria, Rio Grande do Sul; 01 May 1984; Junges, D. leg.; MZSP 027662 •1 shell; Fazenda Itaqueri, Pereque, Rio Grande do Sul; 27 Sep 1968; MZSP 027663 •10 shells; Fazenda Campininha, Mata da Olaria, Mogi Guaçú, São Paulo; 27 Jan 1977; MZSP 027664 •10 specimens preserved in ethanol; Cana Brava, Nova Roma, Goiás; Oct 1952; MZSP 027665 •1 shell; Pirassununga, São Paulo; Schubart, O. leg.; MZSP 027666 •1 ethanol preserved specimen; fazenda Itaquerê, Nova Europa, São Paulo; 01 May 1968; Lenko, K. leg. MZSP 027668 •1 specimen preserved in ethanol; Rio Tapajós, Barreirinha, Pará; 18 Sep 1970; MZSP 027669 •1 specimen preserved in ethanol; Monte Alegre, Amparo, São Paulo; Schubart, O. leg.; MZSP 027670 •2 specimens preserved in ethanol ; Caraguatatuba, between Caraguatatuba and Paraibuna, São Paulo; Sep 1979; Bofi, A.V. leg.; MZSP 029757 •1 shell; Rancho Carmelita, Mogi das Cruzes, São Paulo; Oct 1976; MZSP 029678 •4 specimens preserved in ethanol; Fazenda Itaquerê, Nova Europa, São Paulo, 24 Jan 1964; MZSP 027679 •2 specimens preserved in ethanol; Pará, Santarém, Taperinha; 09 Jan. 1968; Leme, J.L.M. leg.; MZUSP 029680 •1 specimen preserved in ethanol; Rio Doce, Espírito Santo; Jan 1905; Garbe, E. leg.; MZSP 029681 •2 specimens preserved in ethanol; fazenda Ponte Alta, Monte Alegre, São Paulo; Schubart, O. leg.; MZSP 029682 •1 specimen preserved in ethanol; Barueri, São Paulo; 21 Dec. 1965; Lenko, K. leg.; MZSP 029683 •1 ethanol preserved specimen; Marília, São Paulo; 24 Jan 1950; Schubart, O. leg.; MZSP 029685 •1 specimen preserved in ethanol; fazenda Pau d’Alho, Itu, São Paulo; 17 Dec 1960; MZSP 029686 •6 specimens preserved in ethanol; fazenda Pau d’Alho, Itu, São Paulo; 02 Nov 1960; Martins, V. leg.; MZSP 029687 •2 specimens preserved in ethanol; fazenda Pau d’Alho, Itu, São Paulo; Oct 1965; Biasi, P. leg.; MZSP 029688 •1 specimen preserved in ethanol; Piraputanga, Mato Grosso do Sul; Aug 1999; Brisiovit, A.O.; MZSP 034439 •1 specimen preserved in ethanol; Itapetininga, São Paulo; MZSP 049313 •1 specimen preserved in ethanol; Cabo Frio, Rio de Janeiro; 06 Sep 1976; MZSP 049314 •1 specimen preserved in ethanol; same locality; 06 Sep 1976; MZSP 049315 •5 specimens preserved in ethanol; São Paulo, São Paulo; MZSP 050088 •2 shells; Estação Raiz da Terra, São Paulo; 1 Aug 1909; MZSP 050645 •6 shells; Curitiba, Paraná; Apr 1933; Morretes, L.; coleção Lange de Morretes no 1498; MZSP 051615 •1 specimen preserved in ethanol; Mogi das Cruzes, São Paulo; 27 Jan 2007; Fernandez, V. leg; MZSP 083736 •1 specimen preserved in ethanol; Fazenda Tororó, Buerarema, Bahia; 23 Mar 2006; Jahyny, B. leg.; MZSP 086773 •4 shells; Pedro Leopoldo, Minas Gerais; MZSP 107998 •5 shells; Salvador, Bahia; MZSP 108038 •1 ethanol preserved specimen; Fazenda Paraguaçú, Porto Feliz, São Paulo; Feb 1988; MZSP 113624 •6 shells; Rio Grande do Sul, Estrada Velha; 04 Sep 1993; Indrusiak, L. leg.; MZSP 132580 •2 shells; Rio Grande do Sul, , Morro de Santa Antão; Sep 1980; MZSP 132582 •1 shell; Rio Grande do Sul, CISM Eq. Zool.; 29 Dec 1994; MZSP 132583 •4 shells; Santo Antão, Rio Grande do Sul; Sep 1980; Indrusiak, L.; MZSP 132584 •2 shells; Caturrita, Rio Grande do Sul; Aug 1980; Loreto, E. leg.; MZSP 1328585 •3 shells; Sat. de Brito, Rio Grande do Sul; 19 Feb 1992; Indrusiak, L. leg. MZSP 132586 •2 shells; Rio Grande do Sul, Sep 1979; MZSP 132588 •1 shell; same locality as before; 29 Apr 1988; Indrusiak, L.; MZSP 132590 •1 shell; Rio Grande do Sul; Jul 1990; Indrusiak, L. leg.; MZSP 132591 •4 shells; São Paulo; 30 Jul 2009; MZSP 142281 •4 shells and 4 soft parts preserved in ethanol; Rio Grande do Sul, Pelotas; NMNH 637244. **Uruguay** •2 shells; NMNH 530524. **Paraguay** •3 shells; Pasa Yobay; 13 Sep 1978; Delaware Museum of Natural History exped. leg.; ANSP 348185 •4 specimens; Villarrica; FMNH 133267•2 specimens; same collection data as for preceding; FMNH 202413 •3 shells; same collection data as for preceding; MZLU L954/4325 •3 specimens; Martyn, Villarrica; FMNH: 119061 •3 specimens; same collection data as for preceding; FMNH 71601 •1 shell; Guaira department, Villarrica; FLMNH 161257 •preserved specimen; Guairá, Villarrica; Doña Juana stream; MACN 708 •preserved specimen; MACN 10510•preserved specimen; Guairá, Villarrica; MACN 11758 •1 preserved specimen; Canindeyú, indigenous community “ChupaPolu”, 11Km Villa Ygatimú; MACN 38133•1 shell; Villarrica; MCZ: Mala:65665 •1 preserved specimen; San Estanislao; UNLP 8566. **Argentina** •preserved specimen; MACN 26343 •preserved specimen; Misiones; MACN 27289 •preserved specimen; Missiones, Iguazú, Parque Nacional Iguazú; MACN 37738•preserved specimen; Misiones, Iguazú, Parque Nacional Iguazú; MACN 37761. Argentina •1 shell; Salto do Iguazú, argentine side; 01 Aug 1935; Noda, S. leg.; MZSP 016740 •preserved specimen; Misiones, Cerro Corá; 02 Nov 1949; MLP 8563 •preserved specimen; Chaco, Gran Chaco; 1950; MLP 8564 •preserved specimen; Misiones, Posadas; MLP 8565 •preserved specimen; Misiones, Eldorado; UNLP 8567 •preserved specimen; Misiones, Iguazú; MLP 8569.

**Figure 3:**
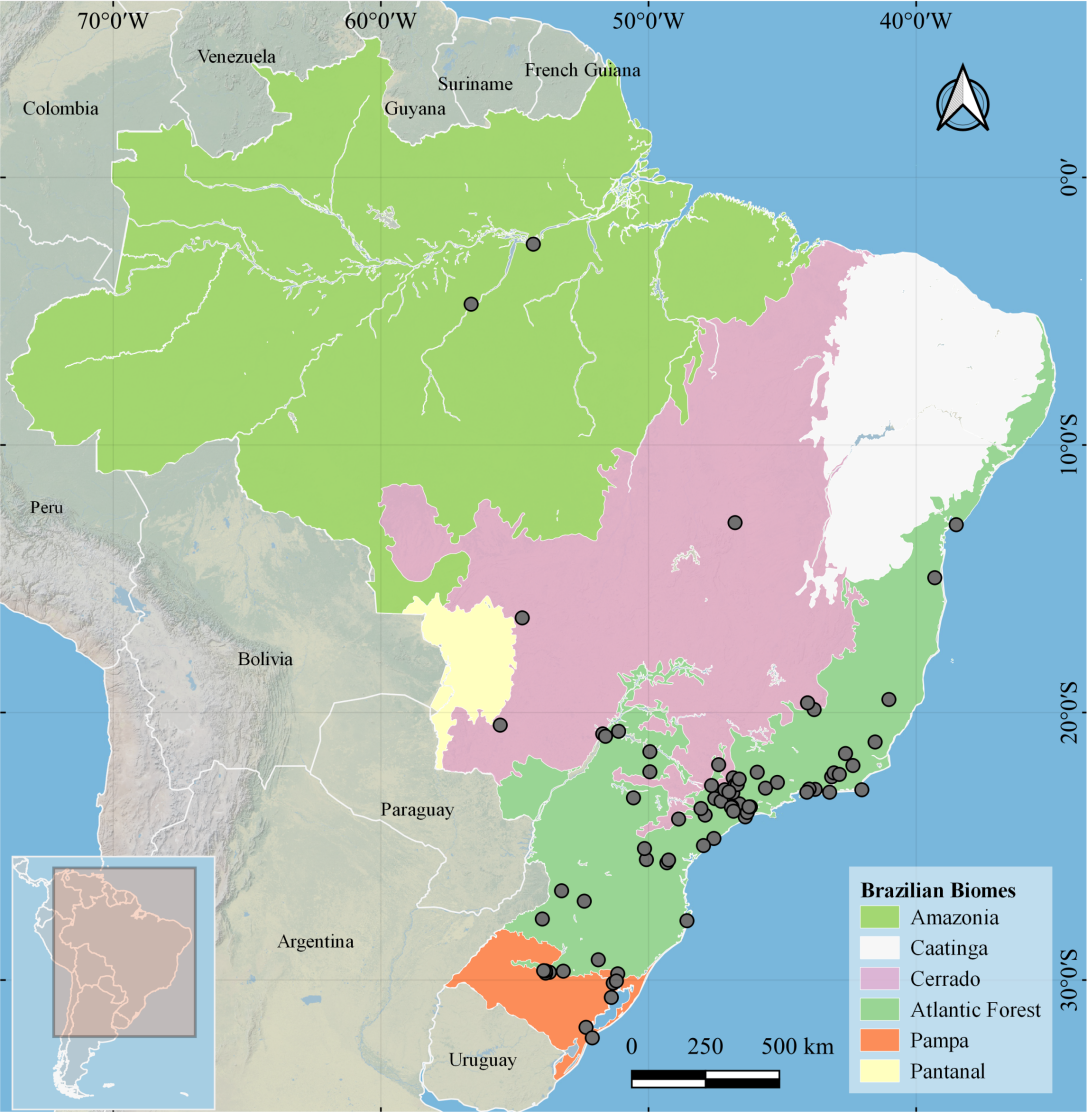
Occurrence records of *Drymaeus (Mesembrinus) interpunctus* in different Brazilian biomes, obtained through an intensive search in the literature, biodiversity databases, and malacological collections.

*Distribution*: Argentina (Parodiz, 1957; Miquel, 1991); Paraguay (Bertoni, 1926; Schade, 1965); Uruguay (Ihering, 1923; Parodiz, 1957). In Brazil, the occurrence records from the literature correspond to localities at Rio de Janeiro (one record), Paraná (three records), Rio Grande do Sul (18) and São Paulo (29) states. The occurrence records for this species correspond to four of the six, biomes from Brazil. The Atlantic Forest was the biome with the highest number of records (Fig. 3). We found one record for Bolivia: Santa Cruz, Buena Vista; Mar 1951; MLP 8570. But after checking the image of the specimen, we confirmed that this is a case of misidentification.

**Figure 4:**
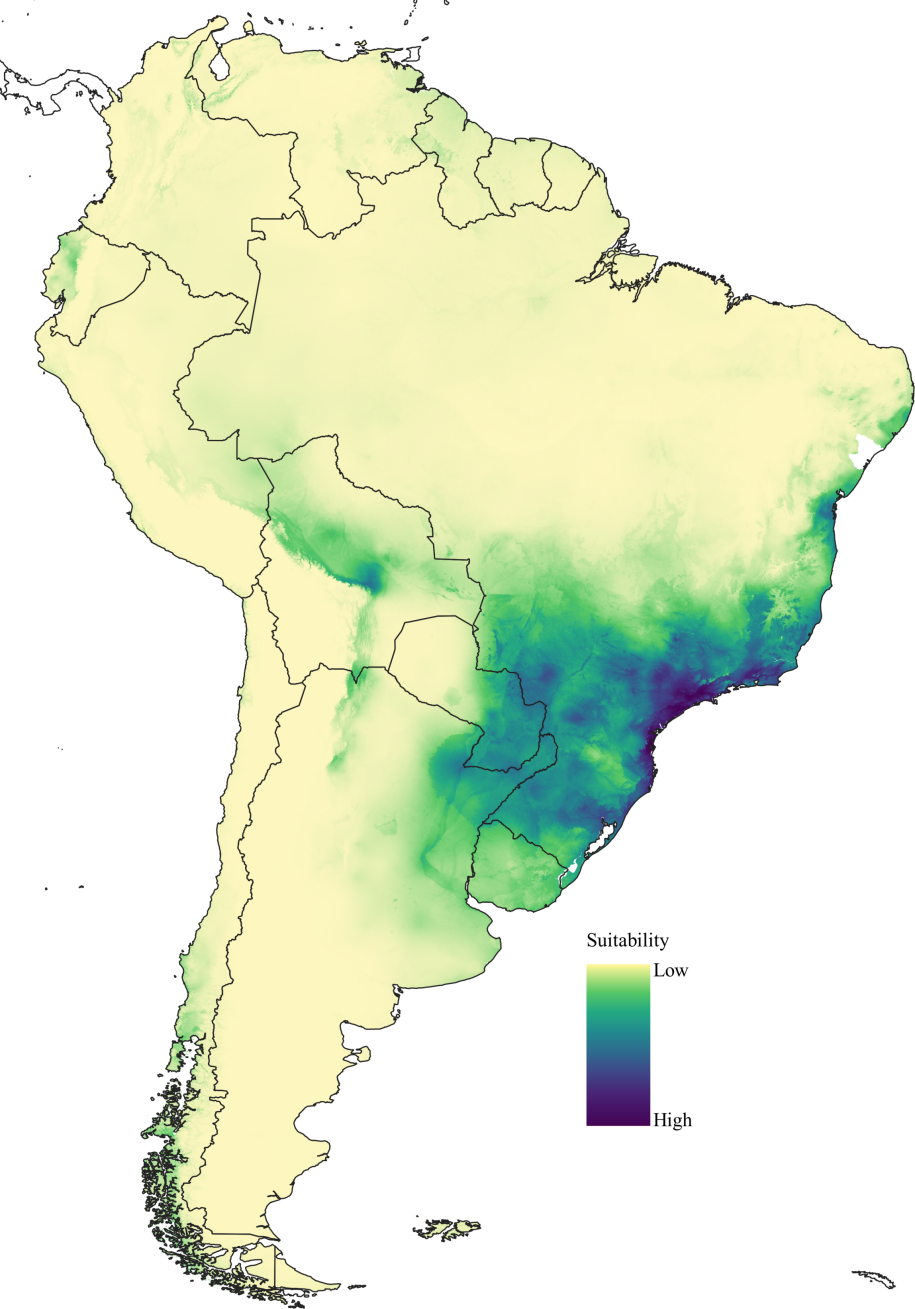
Suitability areas for *Drymaeus (Mesembrinus) interpunctus* obtained from models performed with MaxEnt version 3.3.3k. For niche modelling, nineteen bioclimatic variables and a topographic variable were used as predictors.

*Potential distribution*: AUC calculated from the ROC curve generated for Maxent was 0.932 with a standard deviation of 0.010. The MaxEnt model showed a thin area of high suitability to *Drymaeus* (*Mesembrinus*) *interpunctus* in the southeastern region of Brazil (Fig. 4). This area coincides with the Atlantic Forest biome in the states of Rio de Janeiro, São Paulo, Paraná, and Santa Catarina. The jackknife test of variable importance in MaxEnt showed that Minimum temperature of the coldest month (BIO6) and mean temperature of coldest quarter (BIO11) were the variables that most influenced the development of the model when these variables were used alone (Fig. S1).

### Morphological description

**Figure 5:**
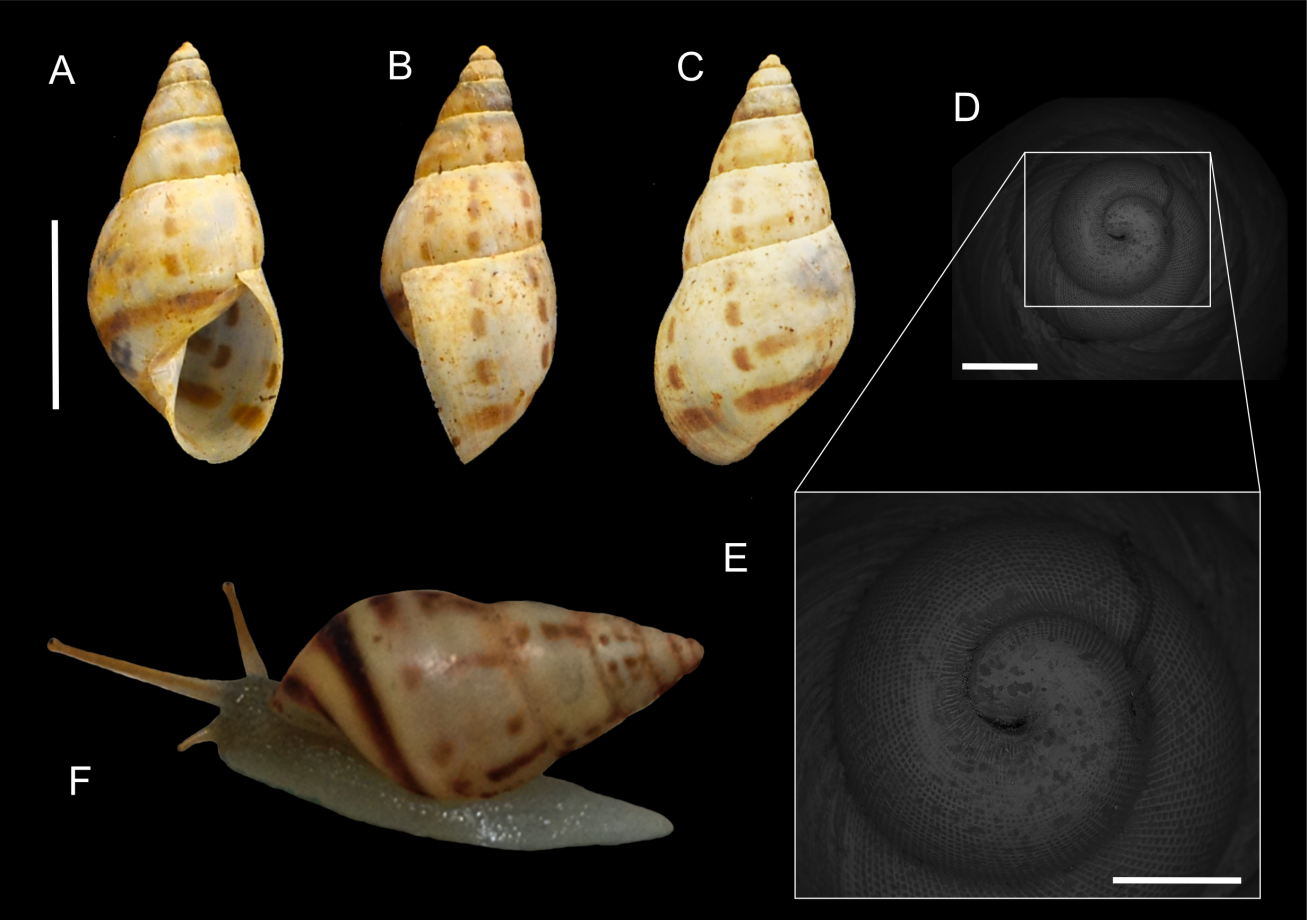
*Drymaeus (Mesembrinus) interpunctus*. (A) Apertural view of specimen from MZSP collection (49313), scale bar = 1 cm. Note coloration pattern. (B) Lateral view, scale bar = 1 cm. (C) ventral view, scale bar = 1 cm. (D) Protoconch in SEM, scale bar = one mm. Note the micro sculpture. (E) Detail of protoconch micro sculpture scale bar = 0.5 mm. (F) Adult specimen showing the coloration pattern of the shell *in vivo*.

*Shell* (Fig. 5): Shell perforate, conic-oblong, thin, very spirally striated; whitish to pale yellow, with widely separated vertical series of brown spots, and two bands on the shell base (Figs. 5A–5C, 5F). Protoconch with a grating sculpture formed by axial riblets and spiral striae (Figs. 5D, 5E). Shell surface smooth with regular and shallow striae. Seven whorls, nearly flat, body whorl rounded at the base, which occupies two thirds of the total shell length. Aperture whitish rather broadly ovate, columella arcuate. Columellar margin narrowly reflexed (Fig. 2A). Outer lip thin, little expanded. Peristome thin and simple (Figs. 5A–5D).

*Head-foot* (Fig. 5): Head-foot almost transparent in living animals, color pale cream (Fig. 5F), with fine tessellation. Foot broad, sole oval, edges rounded in fixed animals, posterior edge almost triangular in live animals (Fig. 5F).

**Figure 6:**
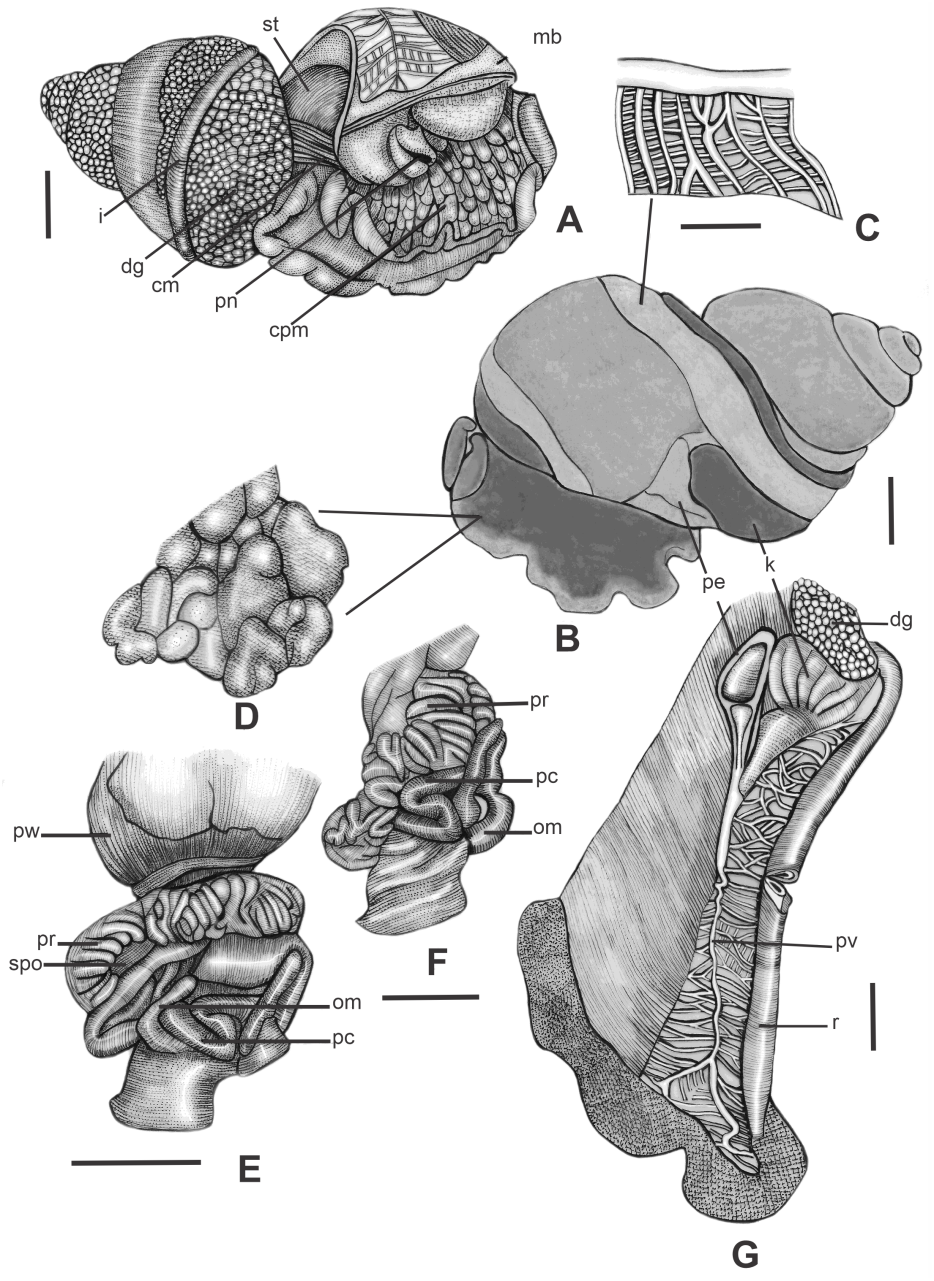
Morphology of pallial and reproductive systems of *Drymaeus (Mesembrinus) interpunctus*. (A) Soft parts out of the shell, scale bar = 5 mm. (B) General morphology of the body showing the position of the kidney and pericardium, scale bar = 5 mm. (C) Detail of the mantle venation, scale bar = 5 mm.** (**D) Detail of the tessellation of the cephalopodal mass, scale bar = three mm.(E, F) part of the reproductive system in anatomical position, scale bar = 5 mm. Legend: i: intestine; cm: columellar muscle; cmpc: columellar margin of the pallial cavity; cpm: cephalopodal mass; dg: digestive gland; k: kidney; mb: mantle border; om: omatophore; pv: pulmonary vein; pc: penial complex; pe: pericardium; pn: pneumostome; pr: prostate; pw: penultimate whorl; r: rectum. spo: spermoviduct; st: stomach.

*Mantle organs* (Figs. 6A–6C; 6G): Mantle edge simple, thick. Rectum narrow, walls thick, running along right edge of pulmonary cavity. Ureter running parallel to rectum (Fig. 6G). Pulmonary cavity short, occupying only the body whorl (Fig. 6B). Kidney and pericardium restricted to middle and left sides, occupying ∼1/3 of pallial roof. Kidney triangular. Anus external to the pallial cavity (Fig. 6A). Pulmonary vein running along entire pallial cavity, oblique in anterior 1/2, running along right edge in remaining 1/2 of pallial roof and bifurcating in the last 1/7 of the right edge (Fig. 6G). Venation very well marked in the right edge of the of pallial roof (Figs. 6A; 6C), between the pulmonary vein and the ureter, and in a restrict part of the right side of the pulmonary vein in remaining 1/2 of the pallial roof, forming a band visible near the suture between the body whorl and the penultimate whorl (Fig. 6B). Venation imperceptible in the other parts of the pallial roof (Fig. 6G). Pericardium located at columellar margin of posterior end of pallial cavity, with approximately the same length as the kidney. Auricle elongate, smaller than the triangular ventricle. Kidney and pericardium occupying ∼1/3 of pulmonary cavity. Kidney with ∼7 lobes, with a slightly longer anterior lobe at the level of the auricle. Pulmonary vein converging to pneumostome region along with renal tube and rectum (Figs. 6A, 6B; 6G).

*Visceral mass* (Fig. 6A). Digestive gland brownish, located along inferior region of penultimate visceral whorl, and filling entire visceral whorls anterior to stomach, including nepionic whorls. Stomach visible at the penultimate whorl. Rectum visible at the body whorl.

**Figure 7:**
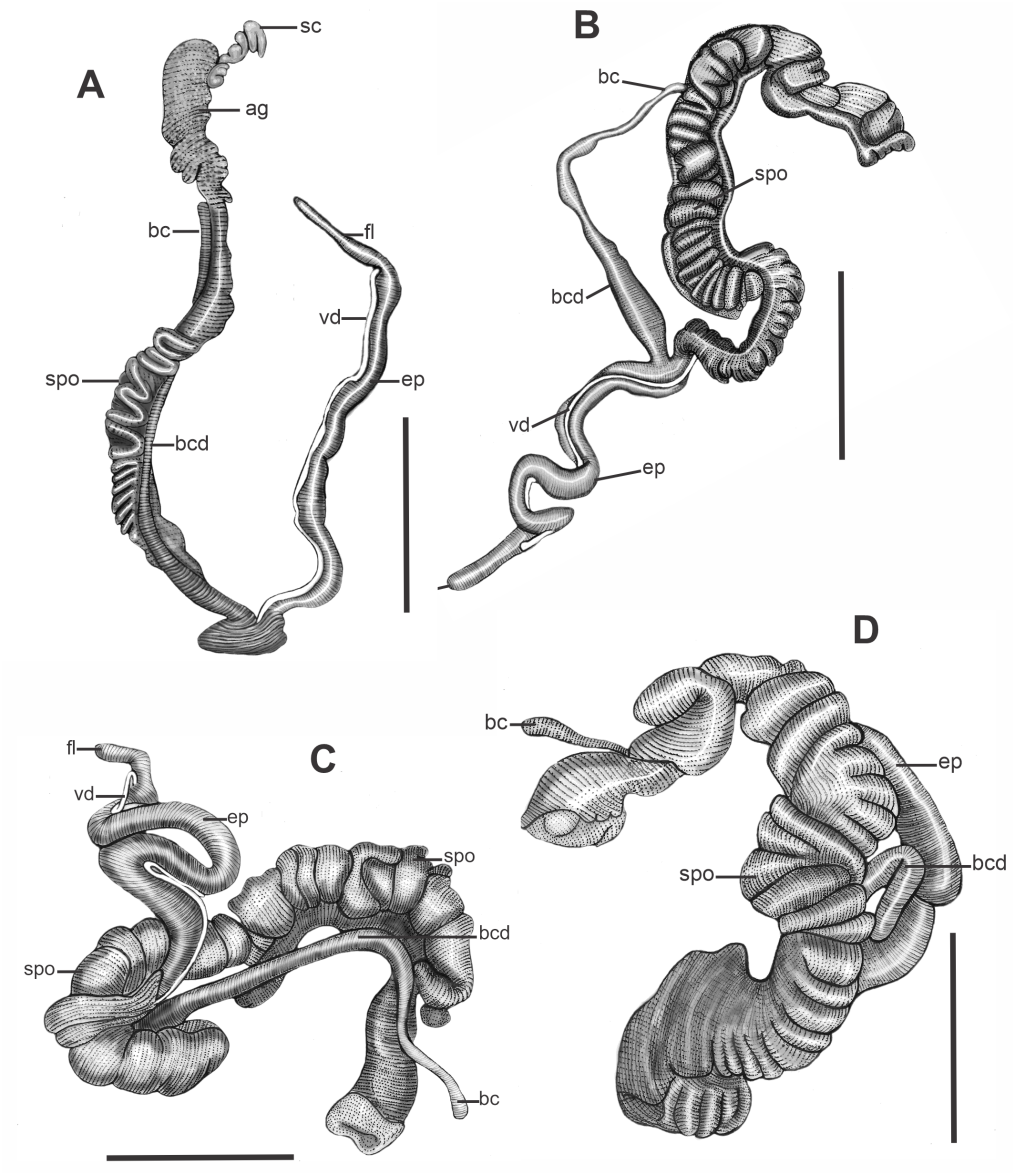
Reproductive system of *Drymaeus (Mesembrinus) interpunctus*. (A) General shape of reproductive system in natural view, scale bar = 5 mm. (B) General shape of reproductive system in ventral view, scale bar = 5 mm. (C) Detail of the anterior portion of the reproductive system showing spermoviduc and penial complex, scale bar = 5 mm. (D) Detail of spermoviduct, scale bar = 5 mm. Legend: ag, albumen gland; bc, bursa copullatrix; bcd, bursa copullatrix duct; ep, epiphallus; fl, flagellum; sc, spermatheca complex; spo, spermoviduct; vd, vas deferens.

*Reproductive system* (Figs. 7A–D): Spermoviduct very long and glandular (Figs. 6A–D), occupying only the body whorl. Bursa copulatrix duct and penial complex with approximately the same length when distended (Fig. 7C). Bursa copulatrix lying at the transition between the spermoviduct and albumen gland (Fig. 7C). Bursa poorly differentiated from the distal portion of the duct. Basal portion of the duct is larger in width than the bursa (Fig. 7D). Prostate occupying the columellar surface of spermoviduct. Penial complex long, sub-cylindrical (Figs. 7A, 7C, 7C), passing under right ommatophore (Fig. 7E). Penis subcylindrical, not covered by a penial sheath. Penis-epiphallus transition undifferentiated. Insertion of the vas deferens subterminal (Figs. 7A; 7D). Flagellum present, subcylindrical, short in relation to the total length of the penial complex (Figs. 7A, 7C, 7D). Nearly 1/10 the length of the penial complex. Vagina very short. Spermatheca complex well differentiated as wide sinuous tube lying at the superior third of the albumen gland (Fig. 7C).

**Figure 8:**
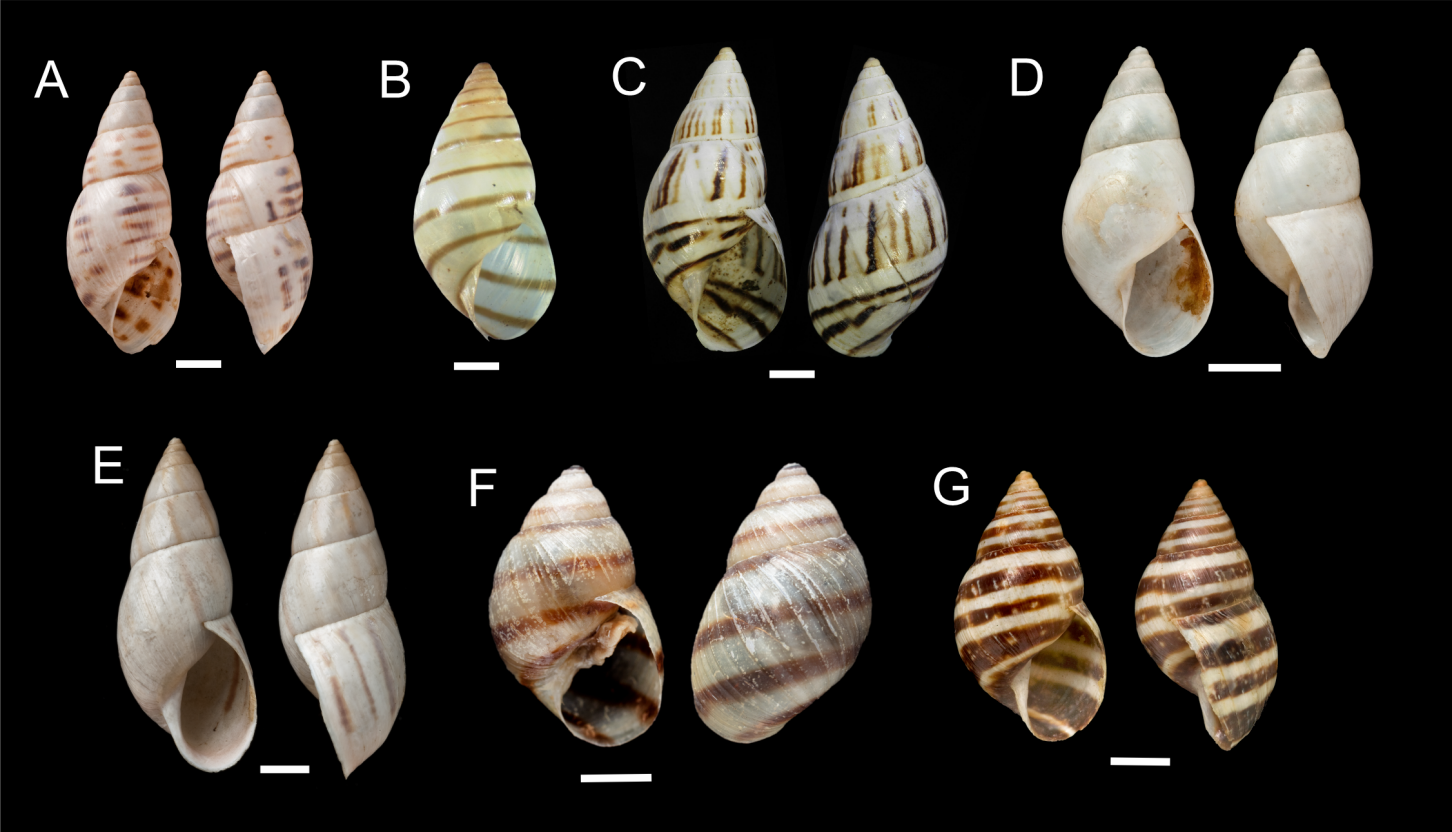
Shells of *Drymaeus (Mesembrinus)* spp. (A) Lectotype of *Drymaeus (Mesembrinus) dutaillyi* NHMUK 1975516, scale bar = 5 mm. (B) Holotype of *Drymaeus (Mesembrinus) lynchi* MACN 1344. (C) Lectotype of *Drymaeus (Mesembrinus) nigrofasciatus* NHMUK 1975542, scale bar = 5 mm. (D) Lectotype of *Drymaeus (Mesembrinus) oreades* NHMUK 1854.12.4.161, scale bar = 5 mm. (E) Lectotype of *Drymaeus (Mesembrinus) puellaris* NHMUK 1975400, scale bar = 5 mm. (F) Lectotype of *Drymaeus (Mesembrinus) roseatus* NHMUK 1975309, scale bar = 5 mm. (G) *Drymaeus (Mesembrinus) rufolineatus* NHMUK 20180262, scale bar = 5 mm.

**Remarks.** *Drymaeus* (*Mesembrinus*) *interpunctus* was described based on specimens collected in Brazil without mention to the exact location of collecting by the author. Later, Ihering (1923) and Morretes (1949) also mentioned this species without specific locality. The new records from databases allowed to expand the known geographic distribution for this species in Brazil and South America. Breure & Eskens (1981) provided a detailed anatomical description of the reproductive system from specimens from, Paraguay, and later, Miquel (1989) provided a description of the shell of one specimen from Argentina. Although the type locality of this species is in Brazil, were it presents a wide geographical distribution, the anatomy of the soft parts of specimens from Brazil was never described before. The anatomy of the reproductive system of the Brazilian specimens analyzed herein mostly corresponds to the description provided by Breure & Eskens (1981), with the exception of the penial sheath, which was not observed in the Brazilian specimens, and also the relative length of the bursa copulatrix duct, which is nearly as long as the spermoviduct in the specimens analyzed herein, while it is about half as long as the spermoviduct in the specimens from Paraguay studied by those authors.

**Table:** 

*Drymaeus (Mesembrinus) dutaillyi* (Pfeiffer, 1857) (Fig. 8A)

**Table:** 

*Bulimus dutaillyi*Pfeiffer, 1857: 390.
*Bulimus dutaillyi*—Pfeiffer, 1859: 470.
*Drymaeus dutaillyi*—Pilsbry, 1901: 158.—Salgado & Coelho, 2003: 161.
*Drymaeus (Leiostracus) dutaillyi*—Morretes, 1949: 152.
*Drymaeus (Mesembrinus) dutaillyi*—Breure, 1979: 118.—Breure & Eskens, 1981: 57—Breure & Ablett, 2014: 65.
*Mesembrinus dutaillyi—*Simone, 2006: 145.

*Original description*: Pfeiffer, 1856. Proc. Zoo. Soc. London 24: 390.

*Illustration*: Breure & Eskens (1981: pl. 7, fig. 2), Simone (2006: fig. 485), Breure & Ablett (2014: fig.22G, L19 iv).

*Type material*: *Lectotype* NHMUK 1975516.

*Type locality*: “Brazils” [Brazil] (Pfeiffer, 1857)

*Museum deposits*. **Brazil** •20 shells; Rio Grande do Sul; Salgado, N. C. ident.; MNRJ 30577.

*Distribution*: We found a record of this species for Rio Grande do Sul.

**Remarks.** This species was described for Brazil without specific type locality. Simone (2006) mentioned “Bra” probably repeating the information in the original description. Breure (1979) designed the lectotype for this species. The record from Rio Grande do Sul represents the first with specific locality for the country.

**Table:** 

*Drymaeus (Mesembrinus) gereti*Ancey, 1901 (Fig. 8B)

**Table:** 

*Drymaeus gereti*Ancey, 1901: 93.
*Drymaeus gereti*—Pilsbry, 1902: 159.
*Drymaeus (Mormus) gereti*—Morretes, 1949: 151.—Oliveira, Rezende & Castro, 1981: 12.—Oliveira & Almeida, 1999: 36.—Salgado & Coelho, 2003: 162.—Simone, 2006: 137.
*Drymaeus (Mesembrinus) gereti*—Breure, 1979: 119.—Breure & Eskens, 1981: 71.

*Original description*: Ancey, 1901, Le Naturaliste 15, 2e Serie, p. 93.

*Illustration*: Simone (2006: fig.452, shell), Breure & Eskens (1981: fig. 223–224, reproductive system).

*Type Material*: Cotype (NMW.1955.158.12853).

*Type locality*: “[Brazil] Prov. De Goyaz” (Ancey, 1901).

*Museum deposits*: **Brazil** •Goiás; ANSP 89850 •1 shell; Ponta Grossa, Paraná; 1 Dec 1939; Lopes, H. S. leg.; MCZ:Mala:171902•Minaçu, Goiás; 23 Jul 2014; MNRJ 5744 •Quirinópolis, Goiás; Feb 1977; Costa, C. J. F. ident.; MNRJ 18827 •2 specimens preserved in ethanol; Jacareci, Bahia; 28 Sep 2006; Salgado. N.C. ident.; MNRJ 31766 •2 shells; Palmeirópolis, near to Mocambo river, Goiás; 29 Nov 2000; Salgado, N.C. ident.; MNRJ 32856•1 shell; Ponta Grossa, Paraná; 1970; Lopes, H. S. leg.; MNRJ 50943 •1 shell; Poços de Caldas, Minas Gerais; Apr 1926; Lopes, H. S. leg.; MNRJ 53635 •2 shells; Bairro do Farol, Maceió, Alagoas; Apr 1955; Cardoso, P. S. leg.; MNRJ 53636 •2 shells; Molhes da Barra do Rio Grande beach, Rio Grande do Sul; May 1975; Lopes, H.S. ident.; MNRJ 56259 [•] 2 shells and 2 specimens preserved in ethanol; Correas, Petrópolis, Rio de Janeiro; Feb 1957; Lopes, H. S. leg.; MNRJ 57609 •Paraíba, Alhandra; 20 Mar 1987; Oliveira, M. col.; MZSP 132135 •1 shell; Bahia; MZSP 027653 •1 ethanol preserved specimen; Goiás, São Domingos; MZUSP 137071 •ethanol preserved specimen; RMNH.MOL 127877 •shell; RMNH MOL 266188.

*Distribution*: This species is endemic of Brazil with records in the states of Espírito Santo, Baixo Guandú (Breure & Eskens, 1981), Corumbá, Mato Grosso do Sul (Oliveira & Almeida, 1999). Alagoas (1 record), Bahia (1), Paraiba (2), Rio de Janeiro (1), Minas Gerais (1), Paraná (1), and Rio Grande do Sul (1) (Fig. 1).

**Remarks.** *Drymaeus* (*Mesembrinus*) *gereti* was described for Goiás and since its description, this species was recorded in others Brazilian states (Breure & Eskens, 1981). According to Wood & Gallichan (2008), the material considered as possible syntype is a specimen from ex collection of Ancey and the confirmation of its validity are a sale list and the cotype label bearing the type locality “Goyaz”.

The occurrence of this species in Brazil is uncertain as its distribution is disrupted, with a wide gap between the records for Trinidad and Tobago and Venezuela and the southernmost records in Minas Gerais, Brazil. However, we have opted to maintain this species in this checklist, as the shell characteristics, the only criterium available for species recognition, of the specimen illustrated in Oliveira & Almeida (1999) correspond to this nominal species.

**Table:** 

*Drymaeus (Memsembrinus) lynchi* Parodiz, 1946 (Fig. 8C)

**Table:** 

*Drymaeus lynchi*Parodiz, 1946a: 1.
*Drymaeus lynchi*—Parodiz, 1946b: 316.—Parodiz, 1957: 25—Solem, 1956:3.—Parodiz, 1962: 439.—Fernandez & Castellanos, 1973: 115.—Oliveira & Almeida, 1999: 38.—Simone, 2006: 139.—Cuezzo, Miranda & Ovando, 2013: 155.—Salgado & Coelho, 2013: 162.—Quintana, 1982: 95.
*Drymaeus (Mesembrinus) lynchi*—Breure, 1979: 121.

*Original description*: Parodiz 1946, Comun. Zool. Mus. Hist. Nat. Monte Video S2 (27) pl.1.

*Illustration*: Parodiz (1946: pl. 2. fig. 3, shell), Simone (2006: fig. 460, shell).

*Type Material*: Holotype. MACN 1344. Paratype. Bolivia MACN 1344-2

*Type locality*: “Pozo Vargas, between Rivers Parapeti and Grande, Bolivia” (*Parodiz, 1946*).

*Museum deposits*: **Brazil** •2 preserved specimens; Corumbá, Mato Grosso do Sul; 19 Dez 1896; FMNH 53067 •6 preserved specimens; Corumbá, Mato Grosso do Sul; FMNH 57058 •1 shell; Corumbá, Mato Grosso do Sul; Santos, S. col.; 1957; CMMPO 1872.

*Distribution*: Paraguay Picuiba, Fortin Nueva Asunción (Quintana, 1982). Argentina (Parodiz, 1957). In Brazil, the records of the species are found in the states of Corumbá, Mato Grosso do Sul (Oliveira & Almeida, 1999). The analysis of the collections databases allowed including a new record for Brazil (Mato Grosso do Sul state) (Fig. 1).

**Remarks.** Salvador, Colley & Simone (2018) synonymized this species with *Drymaeus (Drymaeus) poecilus* (d’Orbigny, 1835) based on the shell pigmentation pattern. In our opinion it is still necessary to examine the anatomy of soft parts and the radula as these two species are ascribed to two different subgenera distinguished by shell traits, anatomical characteristics of the reproductive system, radula and jaw (Breure, 1979; Breure & Eskens, 1981).

**Table:** 

*Drymaeus (Mesembrinus) puellaris* (Reeve, 1850) (Fig. 8D)

**Table:** 

*Bulimus puellaris* Reeve, 1850: 167.
*Bulimus puellaris*—Pfeiffer, 1853:411.—Hupé, 1857:53.—Pfeiffer, 1859:471.—Pfeiffer, 1868:114.—Pfeiffer, 1878:152.—Pilsbry, 1898: 66.
*Drymaeus* (*Mesembrinus*) *puellaris*—Breure, 1979: 123.—Breure & Ablett, 2014:159.

*Original description*: Reeve, 1850, Conch. Iconica, p. 167, pl. 88 fig. 637.

*Illustration*: Reeve (1850: pl. 86. fig. 637).—Pilsbry (1898: pl.11. fig. 8).—Breure & Ablett (2014: Figs. 20H–M, L49iii).

*Imaging*: NHMUK 1975400 lectotype.

*Type Material*: *Lectotype* NHMUK 1975400.

*Type locality*: “Brazil” (Reeve, 1850)

*Museum deposits*: **Bolivia** •1 shell; Corumbá; ANSP 97674. **Brazil** •99 preserved specimens; Botafogo, near to Lagoa Freita [Lagoa Rodrigo de Freitas], Rio de Janeiro; FMNH 26230 •shell; FLMNH 176542 •shell; Botafogo, near Lagoa Freita [Lagoa Rodrigo de Freitas], Rio de Janeiro; FLMNH 109423 •5 preserved specimens; Botafogo, Lagoa Fereita Lagoa Rodrigo de Freitas, Rio de Janeiro; FMNH 119096 •4 preserved specimens; Botabago [Botafogo], Lagoa Fereita [Lagoa Rodrigo de Freitas], Rio de Janeiro; FMNH 125817•3 preserved specimens; FMNH 130711 •8 preserved specimens; Botafago [Botafogo], Lagoa Rodrigo de Freitas, Rio de Janeiro; FMNH 146673 •4 preserved specimens; Para [Pará]; FMNH 77552 •1 shell; Maranon [Maranhão]; MCZ:Mala:26372 •1 shell; MCZ:Mala:64893 •shell; Joinville, Santa Catarina; 01 Jan 1902; Wamardt, W. leg.; MZSP 000618 •1 shell; Franca, São Paulo; MZSP 007761 •2 shells; Meaípe, Guarapari, Espírito Santo; Nov 1993; MZSP112518 •32 shells; Botafogo; RBINS HIST 96 •5 shells; Botafogo , Rio de Janeiro , near Lagoa Rodrigo de Freitas; USMN 408837•2 shells; USMN 57263 •2 shells; Botafogo, Lagoa Rodrigo de Freitas, Rio de Janeiro, on cactus; USMN 589935.

*Distribution*: Bolivia and Brazil (states of Espírito Santo, Rio de Janeiro, São Paulo, and Santa Catarina (Fig. 1).

**Remarks.** The analysis of occurrence records from collection databases allowed including new records for Brazil.

**Table:** 

*Drymaeus (Mesembrinus) oreades* (d’Orbigny, 1835) (Fig. 8E)

**Table:** 

*Helix oreades*d’Orbigny, 1835: 11.
*Bulimus oreades*—d’Orbigny, 1837: 270.—Pfeiffer, 1848: 202.—Pfeiffer, 1853:422.—Pfeiffer, 1859:483.—Pfeiffer, 1868:129.—Reeve, 1850: 93.—Gray, 1854: 15.
*Bulimulus oreades*—Beck, 1837: 65.
*Bulinus sporadicus*—Sowerby, 1833:7.
*Bulimulus (Mesembrinus) oreades*—Martens, 1868: 208 177.—Albers, 1850: 158.
*Drymaeus oreades*—Pilsbry, 1898: 277.—Parodiz, 1957: 25.—Parodiz, 1962: 439.—Fernandez & Castellanos, 1973: 280.—Salgado & Coelho, 2003: 162.—Cuezzo, Miranda & Ovando, 2013: 155.
*Drymaeus (Mormus) oreades*—Morretes, 1949: 150.—Oliveira & Oliveira, 1984: 12.
*Drymaeus (Mesembrinus) oreades*—Breure, 1979: 122.—Breure & Ablett, 2014: 140. —Breure, 2016: 76.
*Thaumastus oreades* —Doering, 1879: 73.
*Mesembrinus oreades* —Simone, 2006: 146.

*Original description*: d’Orbigny, 1835, Mag. Zool., vol.5, p.11.

*Illustration*: d’Orbigny (1837: pl. 31. fig. 11–12), Sowerby (1833: pl.140, fig.78), Reeve (1850: pl. 48. fig. 313), Pilsbry (1898: pl. 44. fig. 95–96), Simone (2006: fig. 489), Breure & Ablett (2014: fig. 54L–M, L43ii).

*Imaging*: NHMUK 1854.12.4.161

*Type Material*: *Lectotype* and *paralectotype* NHMUK 1854.12.4.161

*Type locality*: “de la rive sud du rio Santa-Lucia, dans le environs de San-Roque, province de Corrientes (republique argentine)” (d’Orbigny, 1835).

*Museum deposits*: **Brazil** •2 shells; Piracicaba, São Paulo state; 07 Sep 1897; *v.* Ihering, H. leg.; ANSP 71250 •1 shell; São Paulo state; *v.* Ihering, H. leg.; ANSP 73432 •1 preserved specimen; Bahia; FMNH 31288 •1 shell; Tebas, sítio do sossego, Leopoldina, Minas Gerais; 9 Feb 2009; MNRJ 13369 •1 ethanol preserved specimen; 16 May 2007; Fazenda Triqueda, Coronel Pacheco, Minas Gerais; MNRJ 13370 •1 ethanol preserved specimen; 19 Jan 2007; Fazenda Triqueda, Coronel Pacheco, Minas Gerais; MNRJ 13371 •3 ethanol preserved specimen; 8 May 2007; Coronel Pacheco, Minas Gerais; Junqueira, F.O.; MNRJ 13376 •3 ethanol preserved specimens; 8 May 2007; Coronel Pacheco, Minas Gerais; Junqueira, F.O.; MNRJ 14975 •shell; Piquete, São Paulo; Jan 1896; MZSP 003453 •shell; Est. Alto da Serra, Santo André, São Paulo; MZSP 003455•2 shells; Campinas, São Paulo; Jan 1912; MZSP 007654•2 shells; Campinas, São Paulo; Jan 1912; MZSP 007655 •9 shells; Porto Feliz, São Paulo; 03 Jan 1936; Morretes, L. leg.; MZSP 016772 •2 shells; Lagoa Santa, Minas Gerais; 1971; MZSP 020448 •Ipiranga, São Paulo; the lot is missing; MZSP 027658 •3 preserved specimens; São Paulo; NHMUK1903.4147-9. **Argentina** •1 shell; 16 Jun 1987; NMNH 171434.

*Distribution*: Brazil and Argentina. In Brazil the records correspond to localities in the states of São Paulo, Piracicaba (Ihering, 1923), Ipiranga, Porto Feliz, and Piracicaba, São Paulo state, Brazil (Pilsbry, 1898; Morretes, 1949) (Fig. 7), Minas and Bahia.

**Remarks.** The analysis of occurrence records from collection databases allowed including three new records for Brazil in the states of Minas Gerais. In São Paulo state where this species had already been reported, the number of known localities was increased (4 new records).

**Table:** 

*Drymaeus* (*Mesembrinus) rufolineatus* (Drouët, 1859) (Fig. 8F)

**Table:** 

*Bulimus rufolineatus*Drouët, 1859: 61.
*Drymaeus rufolineatus* —Pilsbry, 1898: 308. —Dutra-Clarke & Souza, 1991: 294.—Simone, 2006: 142.
*Drymeus chevallieri*—Breure, 1976: 113.
*Drymaeus (Mesembrinus) rufolineatus*—Breure, 1979: 123.

*Original description*: Drouët, 1859, Essai sur les mollusques terrestres et fluviatiles de la Guyane française. Paris, Baillière. p. 61.

*Illustration*: Drouët (1859: pl. 1. fig. 10, 11), Pilsbry (1898: pl. 1. fig. 10, 11), Dutra-Clarke & Souza (1991: pl. 2. fig. 2), Simone (2006: fig. 472).

*Imaging*: NHMUK 20180262

*Type Material*: The type material of Drouët is lost.

*Type locality*: “Guyane française, Ilet-laMère” (Drouët, 1859).

*Museum deposits*: **Guiana** •1 shell; MCNB 780200. **French Guiana** •preserved specimen; 1 Apr 2018; Breure, A.S. ident.; NHMUK 20180262. preserved specimen; Cayenne, Camopi, Saut Maripa; 12 Apr 2018; NHMUK 20180263.

*Distribution*: French Guiana. In Brazil, this species has records in the state of Pernambuco, São Lourenço, Estação Ecológica de Tapacurá; Orobó; Serra da Baixa Verde, Águas Belas, Serra Comanati (Dutra-Clarke & Souza, 1991) (Fig. 7).

**Remarks.** According to Breure & Backhuys (2017) the type specimen of the Drouët is considered lost, the only type material available being the paratypes (MNHN 2000-29113; RMNH.MOL.9023) and holotype (MNHN 2000-29176) of *D. chevallieri*, junior synonym of *D. rufolineatus*.

### Species mentioned for Brazil corresponding to misidentifications and unconfirmed records

**Table:** 

*Drymaeus (Mesembrinus) dominicus* (Reeve, 1850)

**Table:** 

*Bulimus dominicus*Reeve, 1850: 172
*Bulimulus hemphilli*—Wright, 1889: 8.
*Bulimulus dominicus*—Fischer & Crosse, 1878: 540.—Strebel, 1873: 94.—Branson & McCoy, 1965.—Thompson, 1967: 249.
*Drymaeus dominicus*—Pilsbry, 1899: 3.—Vanatta, 1908: 101.—Pilsbry, 1905: 39.—Baker, 1922: 10.—Pérez et al., 2008:331.
*Drymaeus (Leptodrymaeus) dominicus*—(Pilsbry, 1946):24.
*Drymaeus (Mesembrinus) dominicus*—(Breure, 1979): 118.—(Breure & Eskens, 1981): 57.—(Thompson, 2011): 117.

*Original description*: Reeve, 1850, Conch. Iconica, p.172, pl. 88 fig. 659. *Illustration*.—Reeve (1850: pl. 88. fig. 659).

*Type material*: not located.

*Type locality*: “S. Domingo; Sallè” (Reeve, 1850).

*Museum deposits*: **Colombia •** 2 shells; New Granada; Swift Collection don.; ANSP 25843. **Brazil** •shell; Viçosa, Alagoas; UF 007692.

*Distribution*: EUA Florida (Wright, 1889; Pilsbry, 1905; Vanatta, 1908; Thompson, 1967). Mexico Veracruz (Strebel, 1873) and Campeche (Branson & McCoy, 1965). Nicaragua (Pérez et al., 2008). Haiti (Pilsbry 1899). Cuba (Pilsbry 1899).

**Remarks.** The known distribution of this species comprises localities in North America (Florida and Mexico), Central America, and Caribbean. The locality associated with the UF 007692 specimen from the Florida Museum of Natural History, labelled as “*Drymaeus dominicus* Brazil, Vicosa [Viçosa], Alagoas”, extends its occurrence for South America, leading a huge gap between its known occurrence area and this new record. We examined digital images of the UF 007692 specimen and its shell traits correspond with most of the characteristics in the textual description given by Reeve. The periostracum of this specimen is not so well preserved, so it is not possible to verify if the band encircling the spire is so minutely interrupted as the drawing of the species provided by Reeve shows. The last whorl is three banded as described. Nevertheless, UF 007692 specimen present differences in shell traits compared to the specimen from Florida imaged by Deisler (2000). Considering that this occurrence record is out of the known distribution area of this species and that type material was not located for comparison, as well as the shell traits of *Drymaeus* species may be easily confounded, we believe thar this record for Brazil may possibly correspond to a case of misidentification.

**Table:** 

*Drymaeus (Mesembrinus) imperfectus* (Guppy, 1866)

**Table:** 

*Bulimus multifasciatus var. imperfectus* (Guppy, 1866): 49.
*Drymaeus imperfectus*—Pilsbry 1899: 19.—Arias (1953): 248.—Oliveira, Rezende & Castro, 1981: 348.—Oliveira & Almeida, 1999: 37.—Simone, 2006: 138.
*Drymaeus (Mesembrinus) imperfectus*—Breure & Ablett, 2014: 197.

*Original description:* Guppy, 1866, Ann. Mag. Nat. Hist. 17 (3) p. 49.

*Illustration*: Pilsbry (1899: pl. 12.; fig. 1, 2, 14, shell), Simone (2006: 138; fig.457, shell).

*Imaging*: NHMUK 1875.28.2

*Type locality*: “…in southern parts of the island” (Guppy, 1866)

*Type Material*: not located.

*Museum deposits*: **Trinidad and Tobago** •4 preserved specimens; ANSP 25863 •preserved specimen; Victoria Co, Moruga Dist. 2.7 km SSE Basse Terre, Trinidad I.; 3 Jun 1994; FLMNH 226974 •1 shell; MCZ:Mala:87139. **Trinidad** •1 preserved specimen; FMNH 94813. **Venezuela** •1 shell; Portuguesa, Acarigua; 1 Jan 1951; MCZ:Mala:172561 •1 shell; Isla de Margarita; 1 Jan 1951; MCZ:Mala:172507. **Brazil** Monjolo, Minas Gerais; 10 Sept 2013; Bichuette, M. col.; MZSP 140455.

*Distribution*: Trinidad and Tobago, and Venezuela, Perijá (Arias 1853)

**Remarks**: We have found records for this species in Brazil in both literature and collections databases. After checking those records, we found that the specimens referred by Oliveira & Almeida (1999), CMMPO 3341 to CMMPO 3350 from Juiz de Fora, Minas Gerais and CMMPO 7310, from Caruaru, Pernambuco, do not correspond to *D. imperfectus*. We were not able to check the record for Monjolo, Minas Gerais, lot MZSP 140455, but considering the distributional gap between the records for Central America and Venezuela and this new record for southern Brazil, we believe that this me be a case of species misidentification.

**Table:** 

*Drymaeus (Mesembrinus) membranaceus* (Phillipi, 1847)

**Table:** 

*Bulimus membranaceus*Philippi, 1847: 126.
*Bulimus membranaceus*—Reeve, 1850: 147—Pfeiffer, 1868: 57—Aubin et al. 1868: 559—Martens, 1893: 252.
*Otostomus (Mormus) membranaceus*—Martens, 1873: 186.
*Otostomus venezuelensis*—Martens, 1893: 224.
*Drymaeus (Drymaeus) membranaceus*—Pilsbry, 1898: 237.
*Drymaeus (Drymaeus) venezuelensis*—Pilsbry, 1898: 312.
*Drymaeus (Mesembrinus) membranaceus*—Breure, 1979: 121.—Breure & Borrero, 2019: 35.

*Original description*: Phillipi, 1847–1850, Abbild. u. Beschreib. neuer oder wenig gekannter Conchylien 2: p. 126.

*Illustration*: Phillipi (1845: pl. 5. fig. 2), Reeve (1850: pl.75. fig. 544), Pilsbry (1898: pl.44. fig.90-91).

*Type Material*: not found.

*Type locality*: Not mentioned by the author in the original description.

*Museum deposits*: **Brazil** •1 preserved specimen; MCZ:Mala:367481 •1 preserved specimen; MCZ: Mala:367483•1 preserved specimen; MCZ: Mala:367483

*Distribution*: Venezuela without specific locality (Breure & Borrero, 2019). Mexico (Pfeiffer, 1868; Martens, 1890), Venezuela Caracas (Martens, 1873).

**Remarks.** According to Breure & Borrero, (2019) the precise occurrence in Colombia remains uncertain. The material housed in MCZ collection would represent the first record in Brazil, According MCZBase (https://mczbase.mcz.harvard.edu/guid/MCZ:Mala:367481), W. G. Binney was who collected that material. Unfortunately, we cannot confirm the validity of these records because the three lots correspond to Microscope slide ledger and no additional material is available.

**Table:** 

*Drymaeus (Mesembrinus) nigrofasciatus* (Pfeiffer in Philippi, 1847 (Fig. 8G)

**Table:** 

*Bulimus nigrofasciatus*—Pfeiffer in Phillipi, 1847: 125.
*Bulimus nigrofasciatus*—Reeve, 1850: 107.—(Strebel, 1873): 47
*Drymaeus nigrofasciatus*—Römer, 1891: 120.—Pilsbry, 1898: 307.
*Drymaeus nigrofasciatus elongatulus*—Pilsbry, 1898: 307.
*Drymaeus (Mesembrinus) nigrofasciatus*—Breure, 1979: 118.—Breure & Ablett, 2014: 131.—Breure & Borrero, 2019: 37.
*Mesembrinus nigrofasciatus*—Simone, 2006: 146.

*Original description*: Phillipi 1847, Abbild. u. Beschreib. neuer oder wenig gekannter conchylien 2: 125.

*Illustration*: Phillipi (1847: pl.5. fig.7), Reeve (1850: pl.55, fig.379), Pilsbry (1898: pl. 50. fig. 96, 97, 98, 99), Simone (2006): fig. 488, shell), Breure & Ablett (2014: Fig. 21 J, L40 v-vi).

*Type Material*: Lectotype and Paralectotypes NHMUK 1975542.

*Type locality*: “vallis Magdalenae Novae Granadae” (Phillipi 1847).

*Museum deposits*: **Venezuela** •1 preserved specimen; original type status: possible syntype; NHMUK 20120333. **Colombia** •1 shell; New Granada; Tryon, G. W., Jr.; ANSP 25834 •2 shells; 14 Jul 1958; ANSP 217109 •shell; FLMNH 106326 •shell; Bogotá; FLMNH 109325 •shell; Bogotá; FLMNH 161271 •shell; FLMNH 161272 •shell; FLMNH 161273 •shell; FLMNH 176960 •shell; FLMNH 176961 •shell; Valle Province, Carmelo; 25 Feb. 1969; FLMNH 176964 •shell; Bogotá Depto; FLMNH 209192 •4 preserved specimens; just NE of Bogotá; FMNH 72764 •2 preserved specimens; Bogotá; FMNH 77560 •2 preserved specimens; just NE of Bogotá; FMNH 77569 •1 preserved specimen; FMNH 94775. •3 preserved specimens; Santa Fe de Bogotá; FMNH 102483 •4 preserved specimens, Sabana de Bogotá, Hernandez lagoon; 17 Apr. 1958; FMNH 114080 •12 preserved specimens; Capitanejo; Aug 1960; FMNH 114081 •1 preserved specimen; Puerto Nariño, Amazonas; 3 Oct 1958; FMNH 114082 •18 preserved specimens; Rocas el Oeste de la Laguna de la Herrera Mun. Mosquera, Sabana de Bogotá; 13 Mar 1960; FMNH 114083 •1 preserved specimen, Ubaté, Cundinamarca; FMNH 115897 •10 shells; Alto de Verea Suscon, 7 km northwest of Sogamosa; 16 Nov 1935; Olson, A.A. leg.; ANSP 166114 •1 preserved specimen; NB 18, Suba (Cundimanarca), Sabana de Bogotá; 19 Sep 1962; FMNH 187577•preserved specimen; Mosquera, La Herrera lagoon; 24 Jun 1981; ICN 4410351•1 preserved specimen; Bogotá, Yomasa; MCZ:Mala:192450. Colombia •1 shell; MCZ:Mala:39284 •1 shell; Bogotá; Dupuis, P. ident.; RBINS HIT 309 •shell; RMNH MOL 106688 •shell; RMNH.MOL.266225. **Venezuela** •1 shell; MCZ: Mala:39300. •shell; Lara 15 Km NE of Duaca; 8 Feb 1989; FMNH 133204. **Peru** •2 preserved specimens; Cerro Prieto 16 mi E of Negritos; FMNH 126191 •1 preserved specimen; Cerro Prieto about 16 mi E from Negritos. **Ecuador** •2 preserved specimens; FMNH 94774.

*Distribution*: Venezuela, Colombia.

**Remarks.** We found records from biodiversity databases of this species in Colombia, Ecuador, Peru and Venezuela. According to Breure & Borrero (2019), this species is restricted to Colombia and the records from Ecuador and Peru are outside the range of the species. Breure & Ablett (2014) commented about the possible misinterpretation of the label of the shell figured by Simone (2006), which is the type specimen of *D. nigrofasciatus* (NHMUK 1975542). The label mention to “Brazil” is probably a mistake that has led to Simone (2006) to consider the occurrence of this species in Brazil. Due to these controversies, we maintain the record of this species in Brazil as uncertain.

**Table:** 

*Drymaeus* (*Mesembrinus*) *roseatus* (Reeve, 1850) (Fig. 8H)

**Table:** 

*Bulimus roseatus*Reeve, 1850: 267.
*Bulimus roseatus*—Sowerby, 1852: 325.—Pfeiffer, 1853: 336.—Mousson, 1869: 176.
*Bulimus dubius*—Pfeiffer, 1851: 257.
*Bulimulus (Drymaeus) roseatus*—Albers, 1860: 212.
*Leiostracus roseatus*—Jousseaume, 1889: 242.
*Drymaeus (Drymaeus) roseatus*—Pilsbry, 1898: 301.
*Drymaeus roseatus*—Salgado & Coelho, 2003: 162.—Simone, 2006: 142.
*Drymaeus (Mesembrinus) roseatus*—Breure, 1979: 123.—Breure & Eskens, 1981: 83.—Breure & Ablett, 2014: 170.

*Original description*: Reeve, 1850, Monograph of the genus *Bulimus*. p. 267; pl.54 fig. 353; b.

*Illustration*: Reeve (1850: pl.54. fig. 353; b).—Pilsbry (1898: pl. 45. fig. 34; 35).—Simone (2006: fig.471).—Breure & Ablett (2014: fig.18 M-N, L52vi).

*Type Material*: *Lectotype*. NHMUK 1975309. *Paralectotypes*. FMNH 54275

*Type locality*: **“**Venezuela**”** (Reeve, 1850).

*Museum deposits*: **Venezuela** •2 preserved specimens; NHMUK 1975310 •1 shell; SNSD 25131.

*Distribution*: Colombia (Simone, 2006) and Venezuela, New Granada (Pfeiffer, 1851). In Brazil, the species was mentioned for state of Amazonas without specific locality (Simone, 2006).

**Remarks.** Salgado & Coelho did not mention specific localities for *D. roseatus* in Brazil, whereas Simone (2006: 142) ascribed the occurrence of this species to Brazil: Amazonas state, and Venezuela. Mousson (1869), who reported on collections made by Wallis in “nördlichen Südamerika”; informed the occurrence of this species for “Amazonas”, however it is unknown if the specific locality was contained in the area corresponding to the state of Amazonas, in our current understanding, or the area correspondent to the tributaries of Amazonas River (which would include Ecuador or Colombia territories as well). Therefore, we maintain the record of this species in Brazil as uncertain.

**Table:** 

*Drymaeus (Mesembrinus) ziegleri* (Pfeiffer, 1847) (Fig. 8I)

**Table:** 

*Bulimus ziegleri* (Pfeiffer, 1847): 113.
*Bulimulus californicus*—Reeve, 1850: 109.—(Pfeiffer, 1853): 422.—(Pfeiffer, 1868): 129.
*Bulimus ziegleri*—Reeve, 1850: 112.—Hupé 1857: 51.—(Pfeiffer, 1848):175.—(Pfeiffer, 1868):115.
*Orthalicus ziegleri*—Carpenter, Gray & British Museum (Natural History), 1855: 177.
*Bulimulus (Liostracus) ziegleri*—Binney & Bland 1869: 193.
*Bulimulus (Drymaeus) californicus*—(Stearns, 1894):165.
*Drymaeus ziegleri*—Pilsbry, 1899: 39.
*Drymaeus (Mesembrinus) ziegleri*—(Breure, 1979): 125.—(Breure & Ablett, 2014): 37.
*Mesembrinus ziegleri*—(Simone, 2006): 146.
*Drymaeus (Drymaeus) ziegleri*—(Thompson, 2011): 111.

*Original description*: Pfeiffer, L. 1846. Proc. Zoo. Soc. London 15: 113.

*Illustration*: Reeve (1850: pl.56. fig. 378), Reeve (1850: pl. 58. fig. 389), Binney & Bland (1869: fig. 336), Pilsbry (1899: pl. 44, fig. 95–96), Breure & Ablett (2014: fig. 54 L-M, L43ii), Simone (2006: fig. 490).

*Imaging*. NHMUK 1975570

*Material*: NHMUK 1975570

*Type locality*: “unkown” (Pfeiffer, 1847).

*Museum deposits*: **Brazil** •3 preserved specimens; NHMUK 1885.10.13.1-3. •2 shells; between Itaipava and Barra de Itapemirim, Espírito Santo; Apr 2014; taken on tropical trees; MZSP 129685 •8 shells; Serra de Sapateira, São Fidelis, Rio de Janeiro; Jun 2009; taken on tropical tree trunk after rain; MZSP 124041 •2 shells; Santa Flora, Santa Maria, Rio Grande do Sul; 04 Jun 1990; Indrusiak, L. leg.; MZSP 132482 •2 shells; Rio Grande do Sul; MZSP 132588. **Argentina** •1 shell; Misiones; 26 Feb 2018; MZSP 136468.

*Distribution*: EUA: California, (Reeve, 1850). Mexico, (Pilsbry, 1899). In Brazil: São Paulo, Rio de Janeiro and Rio Grande do Sul states l, São Paulo, Piracicaba (Simone, 2006) (Fig. 1).

**Remarks.** Simone (2006) figured specimens from lot 1975570 as “possible syntypes” from Brazil, as *Mesembrinus ziegleri* validating the presence of this species in Brazil. However, according to Breure & Ablett (2014), Pfeiffer would has described this species based on material from an unknown locality in northwestern Mexico. Also, they mentioned the possibility that the original material may has been lost and the label “Brazils” was mistrusted by Pfeiffer as he may have seen material from other sources. The analysis of material MZSP correspond to *D*. (*M*.) *interpunctus* and not *Drymaeus (Mesembrinus) ziegleri*.

## Discussion

In the present study, from the analysis of occurrence records of thirteen species of *Drymaeus*, subgenus *Mesembrinus*, we have confirmed the occurrence of seven species in Brazil; the records of six species, *D. dominicus*, *D. imperfectus*, *D. membranaceus*, *D. nigrofasciatus*, *D. roseatus*, and *D. ziegleri*, in both the literature and biodiversity databases being considered doubtful. The records of *Drymaeus imperfectus*, *D*. *dominicus*, and *D. ziegleri* for Brazil, obtained from malacological collections, fall outside the known distribution of these species, with huge gaps between the confirmed and the new occurrence localities. Thus, we believe that these records for Brazil most probably correspond to other species. We were able to check the images of the shells of *D. imperfectus* and *D*. *dominicus* specimens. However, despites the similarities in shell shape and color pattern, the significant gap between the known distribution of these species and the new records available from museum specimens justifies our decision to consider the records for Brazil as uncertain until new data from field collections is available. Interspecific similarity in shell traits between species of *Drymaeus* from different geographical areas is not uncommon and, in those cases, it is necessary to provide anatomical and molecular data to provide a more reliable delimitation of the species. The Colombian species *D. nigrofasciatus* is mentioned to occur in Brazil (Simone, 2006). However, the shell of the type specimen figured by Simone (2006) bears a disputed label according to Breure & Ablett (2014), who warned for misinterpretation. *Drymaeus roseatus* also considered by Simone (2006: 142) as part of the Brazilian malacofauna, was considered to occur in Amazonas state. However, the exact itinerary associated to the collections made by Wallis in “nördlichen Südamerika” and reported by Mousson (1869), is unknown. Wallis travelled in “verschiedene Theile Columbias, Ecuadors und von Amazonas” and Mousson reported the species for Amazonas, however it is unknown if this was the state of Amazonas in current understanding, or the river Amazonas. In the latter case it is unclear whether this was in Brazil, Ecuador or Colombia (ASH Breure; personal communication).

In the present study most of the species of *Drymaeus*, subgenus *Mesembrinus*, were represented in the consulted malacological collections by dry specimens (shells). Only the species *D.* (*M.*) *gereti*, *D.* (*M.*) *puellaris* and *D.* (*M.*) *interpunctus* were represented by specimens preserved in ethanol. We found some records of *D*. (*M*.) *oreades* specimens preserved in ethanol from the malacological collection of Museu Nacional do Rio de Janeiro; but unfortunately, this material was lost during the fire that took place in September of 2018.

Most of the species are represented by scarce material in a few malacological collections; being predominantly housed in malacological collections from North America and Europe. In some cases these records correspond to type material [*i.e.*: *D.* (*M.*) *dutaillyi* (two records, including one record of a lectotype, in two collections), *D.* (*M.*) *lineolatus* (2 records in 2 collections), *D.* (*M.*) *lynchi* (4 records, including one record of the holotype and one record of a paratype, in 2 collections), *D.* (*M.*) *membranaceus* (3 records in one collection), *D.* (*M.*) *roseatus* (4 records, including one record of a lectotype and one record of paralectotypes, in 4 collections); *D.* (*M.*) *rufolineatus* (4 records in 2 collections); *D.* (*M.*) *ziegleri* (7 records in 2 collections)]. This may indicate that these species have not been recollected over time. Alternatively, the scarcity of records obtained herein may also be a result of the deposit of specimens in local, not computerized collections, as for *D.* (*M.*) *dominicus*, for example, we have obtained a reasonable number of occurrence records from the literature. In any case, the absence of specimens suitable for anatomical studies in the main malacological collections worldwide, renders challenging any attempt to revisit these species to provide new morphological information. The scarcity of specimens with preserved soft parts, along with the virtual absence of recollection of live specimens also makes it difficult to compare the anatomies and to obtain DNA samples with the aim of establishing the long-needed anatomy and molecular-based operational criteria for species delimitation.

Breure & Eskens (1981) redescribed the species of *Drymaeus* providing new information on the anatomy of the reproductive system of species of the subgenus *Mesembrinus*. It is important to note that even with the available information on the anatomy of the soft parts of *Drymaeus* provided by previous authors (Pilsbry, 1898; Breure, 1979; Breure & Eskens, 1981), which have been demonstrated to have diagnostic value, species are still being described and synonymized based uniquely on shell morphology and color pattern.

Herein, the species with greater number of records in the malacological collections were *D.* (*M.*) *interpunctus* (128 records), *D.* (*M.*) *imperfectus* (20 records), *D.* (*M.*) *gereti* (16 records), *D.* (*M.*) *nigrofasciatus* (40 records), *D.* (*M.*) *puellaris* (19 records) and *D.* (*M.*) *oreades* (15 records). These species were also represented in 5 to 13 different malacological collections [ie.: *D.* (*M.*) *interpunctus* (13 collections), *D.* (*M.*) *imperfectus* (6), *D.* (*M.*) *gereti* (5), *D.* (*M.*) *nigrofasciatus* (9), *D.* (*M.*) *puellaris* (7) and *D.* (*M.*) *oreades* (8)]. Interestingly, the better representativeness of these species in the collections is not necessarily linked to a broader geographic distribution. For example, the species *D.* (*M.*) *dominicus*, which is poorly represented in the consulted malacological collections, has the widest distribution amongst the species analyzed herein, occurring in seven different countries of Central America and South of North America; the occurrence records for this species being obtained mostly from the literature.

The distribution maps showing the biomes occupied by the species of the genus *Drymaeus*, subgenus *Mesembrinus* showed that all the Brazilian biomes included at least three species. *Drymaeus* (*Mesembrinus*) *interpunctus* is the only species present in all six Brazilian biomes, followed by *D.* (*M.*) *gereti* recorded in the biomes Atlantic Forest and Cerrado. Most of the records were obtained for the Atlantic Forest and Cerrado biomes, which are amongst the world’s biodiversity “hotspots”, with the highest endemism (Mittermeier et al., 2011). The Atlantic Forest, however, was exploited for centuries and only a small proportion of the original forest covering remains nowadays. Similarly, the Cerrado have recently undergone destruction by fires. Thus, species occurring in both biomes should be continuously monitored, and their conservation status accessed, with the aim of assuring their protection.

Herein, *D.* (*M.*) *interpunctus* was recorded for all Brazilian biomes. Interestingly, the results of niche modeling showed a thin area of high suitability to this species in South and Southeastern Brazil corresponding to the Atlantic Forest biome in the states of Rio de Janeiro, São Paulo, Paraná, and Santa Catarina; and a vast area of moderate suitability including the Atlantic Forest, Pantanal, Cerrado, and Pampa biomes. We have found two isolated records for the species in Pará state, North Brazil, corresponding to a low suitability area. We do not reject the possibility of misidentification, as we could not check the species identity of the museum specimens associated to these records. However, this result may possibly be explained by the introduction of this species outside its suitable range, as *D. mesembrinus* is often found in urban areas, which may facilitate its introduction by means of human activity.

Our results may indicate that *D.* (*M.*) *interpunctus* present a niche breadth that might favor its occurrence in a range of different biomes including less suitable areas (Sexton et al., 2017). This would partly explain the contrasting wide geographic extension of this species when compared to other *Drymaeus* species in Brazil. Unfortunately, there is scarce information on this and the other species of *Drymaeus* analyzed herein. Critical knowledge gaps are particularly concerning the ecology and life history traits of these species, for example, temperature ranges and other physical factors that *Drymaeus* species can tolerate, the optimal climatic conditions for their reproduction and growth, their feeding habits, the variety of food items they can eat, and the diversity of microhabitats they can inhabit. Thus, although we are not able to characterize these species in relation to the level of ecological specialization, our results on suitable areas and the potential distribution of *D.* (*M.*) *interpunctus* suggests that it may represent a generalist lineage. Although the number of localities compiled for the remaining species did not allow ecological niche modeling, the small number of occurrence records from the literature and the limited representation in malacological collections observed may indicate that they may have restricted ranges and they may fall into the IUCN categories of vulnerable or endangered (Breure & Vega-Luz, 2020). If the more restricted distributions of these species are a consequence of being more ecologically specialized, this may have consequences over their conservation status, considering the pressures on species to adapt in response to environmental shifts, particularly those related to climate change (Sexton et al., 2017).

## Conclusion

1- Considering the small number of records obtained for most of the species herein analyzed, and that most of the specimens were collected several decades ago, it is probable that these species are rare.

2- The restricted ranges observed for most of the species of *Drymaeus*, subgenus *Mesembrinus*, analyzed herein, associated with historical and recent events leading to habitat destruction may indicate that they are of conservation concern, probably falling into the IUCN Categories of vulnerable or endangered.

3- The records obtained for *Drymaeus* (*Mesembrinus*) *dutaillyi* and *Drymaeus* (*Mesembrinus*) *lynchi* corresponded uniquely to type specimens, indicating the urgent need to perform field surveys to try to obtain live specimens and to access their conservation status.

4- The disparities in geographic range and occurrence records available for different species of *Drymaeus*, subgenus *Mesembrinus* in Brazil, may be associated to differences in the level of ecological specialization and climatic niche breadth, which may ultimately have consequences on their capability to respond to environmental changes. However, the low number of occurrence records available for these species impairs the development of ecological niche modeling for a greater set of taxa, pointing out the need to recollect these species and to obtain ecological information on them.

## Supplemental Information

10.7717/peerj.16037/supp-1 Supplemental Information 1 Ecological niche model performance accessed through the receiver operating characteristic (ROC)

10.7717/peerj.16037/supp-2 Supplemental Information 2 Ecological niche model performance accessed through the method of the area under the curve (AUC) The AUC represents the probability for the model to score a presence site (test locality) higher than a random background site.

10.7717/peerj.16037/supp-3 Supplemental Information 3 Occurrence records for species of Drymaeus, subgenus Mesembrinnus and associated data

10.7717/peerj.16037/supp-4 Supplemental Information 4 Source, geographical representativity, and taxonomic identity confirmation of the occurrence records compiled for thirteen species of *Drymaeus*, subgenus *Mesembrinus*

10.7717/peerj.16037/supp-5 Supplemental Information 5 Occurrence localities for *Drymaeus Mesembrinus* interpunctus.

## Institutions Acronyms

ANSP: Academy of Natural Sciences of Philadelphia
BMSM: Bailey-Matthews National Shell Museum
CMMPO: Coleção Malacológica Maury Pinto de Oliveira
FMNH: Field Museum of Natural History
ICN: Universidad Nacional de Colombia
LMD: Löbbecke Museum of Dusseldorf
MACN: Museo Argentino de Ciencias Naturales Bernardino Rivadavia
MCZ: Museum of Comparative Zoology
MHNG: Muséum d’Histoire Naturelle, Geneva
MNRJ: Museu Nacional do Rio de Janeiro
MZB: Museu de Ciències Naturals de Barcelona
MZLU: Lund Museum of Zoology
MZSP: Museu de Zoologia da Universidade do Estado de São Paulo
RBINS: Royal Belgian Institute of Natural Sciences
RMNH: Rijksmuseum voor Natuurlijke Historie
SNSD: Collection Malakologie –Senckenberg Society for Nature Research
UF: Florida Museum of Natural History
MLP: Museo de La Plata
ZMB: Museum für Naturkunde, Berlin

## Additional Information and Declarations

### Data Availability

The following information was supplied regarding data availability: The raw data is available in the Supplementary Files.
