# Supplementary figures and images for: Species catalogue of Drymaeus (Mesembrinus) Alberts, 1850 (Gastropoda: Bulimulidae) from Brazil and new data on morphology and distribution of Drymaeus (Mesembrinus) interpunctus (Martens, 1887)
Source: PeerJ. 2023 Oct 6;11:e16037. doi: 10.7717/peerj.16037 (PMC10561649; doi:10.7717/peerj.16037)

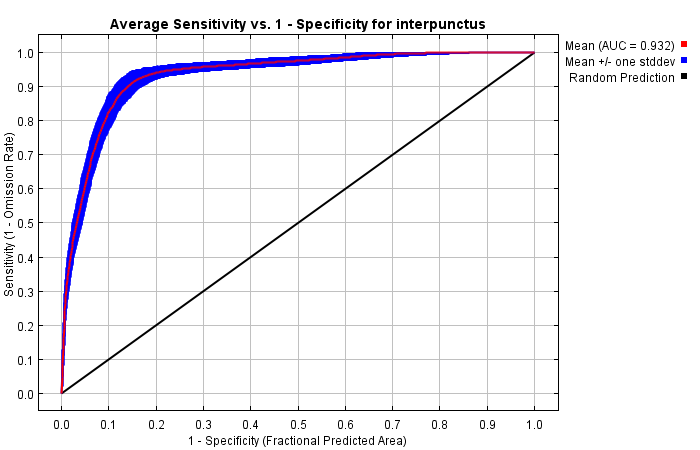

Supplement: Supplemental Information 1 [file peerj-11-16037-s001.png]

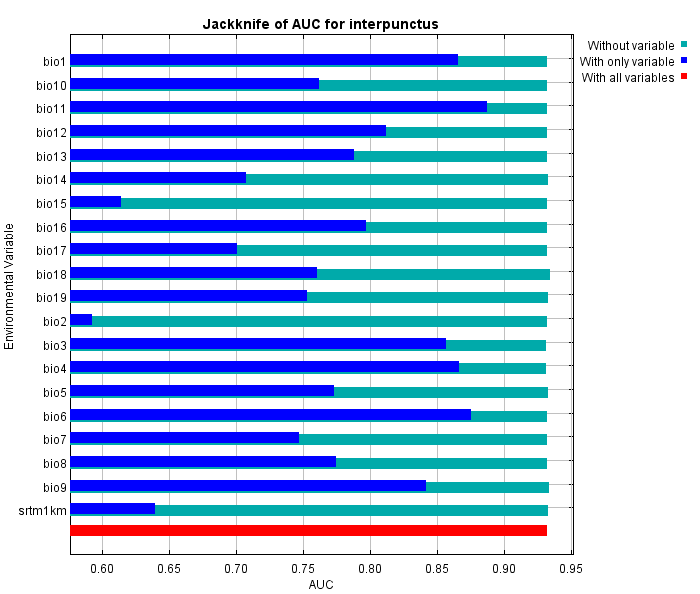

Supplement: Supplemental Information 2 — The AUC represents the probability for the model to score a presence site (test locality) higher than a random background site. [file peerj-11-16037-s002.png]
