# Supplementary material for: Species catalogue of Drymaeus (Mesembrinus) Alberts, 1850 (Gastropoda: Bulimulidae) from Brazil and new data on morphology and distribution of Drymaeus (Mesembrinus) interpunctus (Martens, 1887)
Source: PeerJ. 2023 Oct 6;11:e16037. doi: 10.7717/peerj.16037 (PMC10561649; doi:10.7717/peerj.16037)

Supplemmentary Table S1. Source, geographical representativity, and taxonomic identity confirmation of the occurrence records compiled for thirteen species of *Drymaeus*, subgenus *Mesembrinus*.


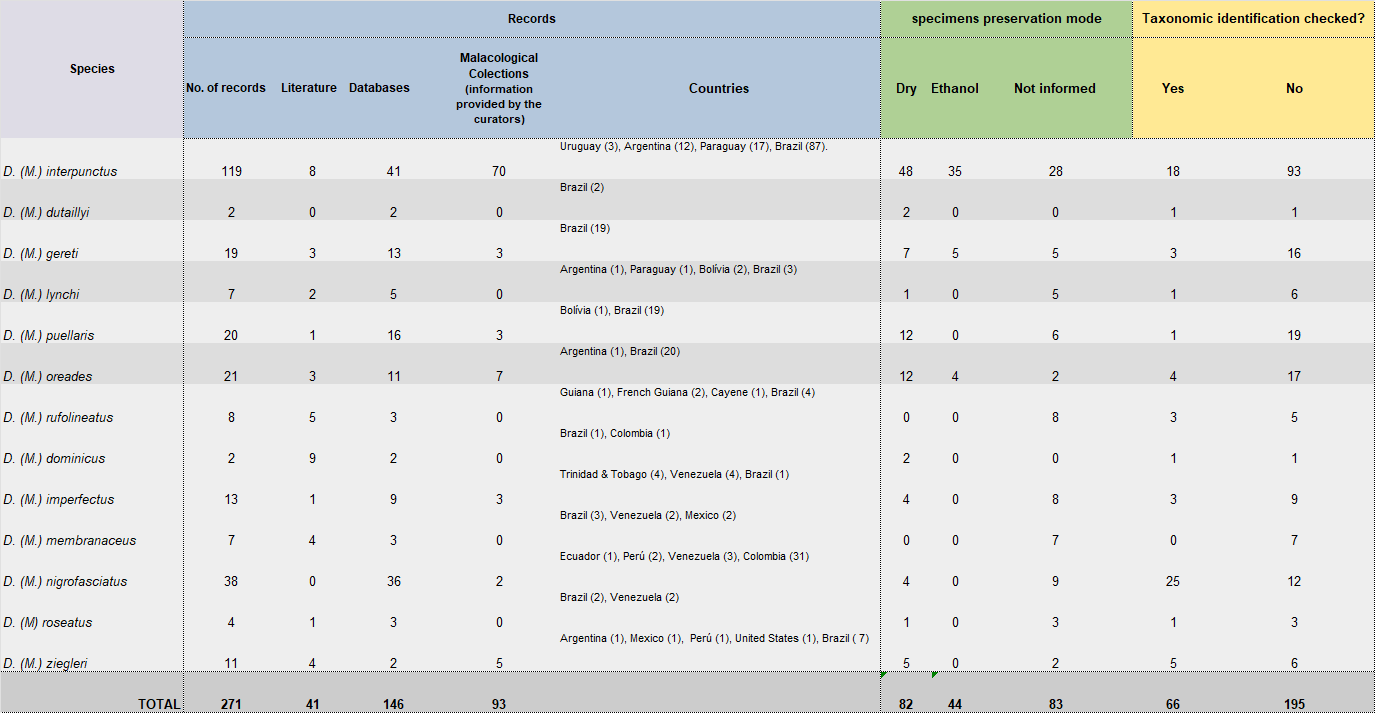

Supplement: Supplemental Information 4 [file peerj-11-16037-s004.docx]
